# Real-World Sacubitril/Valsartan Adoption and Post-Initiation Dose Reassessment and Titration in Heart Failure with Reduced Ejection Fraction: An Explanatory Sequential Mixed-Methods Study from Northern Saudi Arabia

**DOI:** 10.3390/jcm15208001

**Published:** 2026-10-15

**Authors:** Reham Olayan Alonayzan, Alhareth Alsa’d, Zainab Zakaraya, Marwa A. Malouh, Anas Abed, Hayam A. AlRasheed

**Affiliations:** 1 Department of Biopharmaceutics and Clinical Pharmacy, Faculty of Pharmacy, Al-Ahliyya Amman University, Amman 19328, Jordan; rehamalonayzan@amchg.com (R.O.A.); a.alsad@ammanu.edu.jo (A.A.); m.abumalouh@ammanu.edu.jo (M.A.M.); a.abed@ammanu.edu.jo (A.A.); 2 Department of Pharmacy Practice, College of Pharmacy, Princess Nourah bint Abdulrahman University, P.O. Box 84428, Riyadh 11671, Saudi Arabia; haalrasheed@pnu.edu.sa

**Keywords:** sacubitril/valsartan, angiotensin receptor–neprilysin inhibitor, HFrEF, guideline-directed medical therapy, dose titration, prescribing quality, implementation science, mixed methods, multidisciplinary care, Saudi Arabia

## Abstract

**Background/Objectives**: Sacubitril/valsartan is a foundational therapy for heart failure with reduced ejection fraction (HFrEF), but real-world implementation extends beyond initiation to laboratory monitoring, follow-up, dose reassessment, and documentation of reasons for sub-target therapy. We evaluated this prescribing pathway in a regional Saudi heart-failure service. **Methods**: This explanatory sequential mixed-methods study included 296 adults with HFrEF (left ventricular ejection fraction ≤40%) treated at a public referral hospital in Al-Qurayyat, Saudi Arabia, during 2020–2025. Quantitative analyses described active prescription, calendar-period variation, selected physiological caution features among non-users, initiation pathways, early monitoring, follow-up, escalation attempts, and dose status. Fifteen clinicians participated in semi-structured interviews analyzed using the Framework Method and the updated Consolidated Framework for Implementation Research; reporting followed COREQ. **Results**: Sacubitril/valsartan was active in 239/296 patients (80.7%). Among 57 non-users, 41 (71.9%) had at least one selected physiological caution feature. In 221 treated patients with ≥6 months of follow-up, 75.1% completed renal-function/potassium reassessment within 14 days, 67.9% had follow-up within six weeks, and 60.6% had an escalation attempt within 90 days. Current target-dose use was 16.7%, and 21.7% had reached target at any point. Of 116 patients currently receiving 24/26 mg twice daily, 91 had never been documented above the lowest dose, and 25 had previously reached a higher dose. Reasons for current lowest-dose therapy were clinical/physiological in 49, health-system/access-related in 5, patient preference/adherence-related in 4, and undocumented in 58. Qualitative findings emphasized monitoring continuity, explicit role ownership, appointment access, dose availability, and communication. **Conclusions**: High ARNI uptake did not ensure complete post-initiation dose reassessment and titration. The clearest measurable gap was uncertainty around current lowest-dose therapy when no reason or reassessment plan was documented; this represents a documentation/reassessment gap rather than proof of inappropriate care. Clinical audit should distinguish target-dose attainment, clinically limited sub-target dosing, active titration, return to a lower dose after prior escalation, access-related barriers, patient preference/adherence, and unresolved sub-target therapy.

## 1. Introduction

Heart failure (HF) remains a major global cause of morbidity, premature mortality, recurrent hospitalization, and healthcare expenditure. More than 60 million people are estimated to live with HF worldwide, but its clinical presentation and treatment environment vary substantially across regions [1,2]. Saudi Arabia has a particularly important cardiometabolic burden, with high prevalences of diabetes, hypertension, obesity, dyslipidemia, and premature ischemic heart disease. Registry data have also shown that Saudi patients with HF often present at a younger age than patients in many Western cohorts, increasing the social and economic consequences of chronic disease [3,4,5].

Treatment of heart failure with reduced ejection fraction (HFrEF) has changed substantially during the past decade. Contemporary American, European, and Saudi guidance supports early use of four foundational drug classes: an angiotensin receptor–neprilysin inhibitor (ARNI), an evidence-based beta-blocker, a mineralocorticoid receptor antagonist (MRA), and a sodium–glucose cotransporter-2 (SGLT2) inhibitor [6,7,8,9]. Sacubitril/valsartan demonstrated superiority to enalapril in PARADIGM-HF and can be initiated safely in selected stabilized patients during or soon after hospitalization, as supported by PIONEER-HF and TRANSITION [10,11,12]. Dose-escalation studies and contemporary pathways emphasize progression toward target or maximally tolerated doses with close blood-pressure, renal-function, and potassium surveillance [9,13,14]. The rapid expansion of SGLT2 evidence across the heart-failure spectrum further illustrates the pace of change in guideline-directed pharmacotherapy [15,16,17].

The existence of evidence and guidelines does not guarantee implementation. Real-world registries have documented under-prescription, delayed initiation, low target-dose attainment, and treatment discontinuation across multiple settings [18,19,20,21,22,23,24]. Recent implementation statements emphasize that potential therapeutic inertia is not a single clinician decision; it is frequently the cumulative consequence of fragmented follow-up, uncertainty about responsibility, incomplete monitoring, competing comorbidities, limited appointment access, and weak systems for recalling patients below target dose [25,26]. Conversely, pharmacoutilization studies demonstrate that rapid titration and high persistence are achievable when structured pathways are available [27].

The distinction between adoption and post-initiation dose reassessment and titration is central. Adoption refers to whether a patient receives sacubitril/valsartan. Post-initiation dose reassessment and titration is a longitudinal process that includes selecting the starting dose, managing ACE-inhibitor washout when applicable, arranging renal-function and potassium monitoring, reassessing blood pressure and symptoms, escalating or reducing the dose, and documenting the reason for persistent sub-target therapy. Importantly, an intermediate dose should not automatically be considered “optimized”: it may represent active titration, a clinically limited maintenance dose, or unresolved sub-target persistence. Likewise, a low dose may be appropriate when intolerance is documented but potentially remediable when no reassessment occurs.

Saudi real-world studies have mainly examined clinical characteristics, safety, ventricular function, readmission, or effectiveness among patients already receiving sacubitril/valsartan [28,29,30], while broader reviews have summarized effectiveness and safety across settings [31]. These studies do not fully describe the complete implementation pathway: who receives treatment, the clinical context of non-prescription, how initiation and early monitoring are organized, how rapidly doses are revisited, and whether sub-target dosing reflects documented clinical limitation or incomplete reassessment. This distinction is increasingly important because contemporary real-world studies, including the REAL.IT program, demonstrate that target-dose attainment varies markedly between systems [27,32]. Evidence is especially limited in geographically peripheral Saudi settings, where travel, specialist access, laboratory workflows, and pharmacy processes may differ from high-volume metropolitan programs.

Al-Qurayyat is a northern border region located far from the major tertiary cardiac hubs of Riyadh and Jeddah. The regional public hospital provides cardiology, internal medicine, inpatient, and ambulatory services to urban and surrounding communities. In this setting, high medication availability may coexist with constrained clinic capacity, variable access to laboratory testing, and dependence on longitudinal local care. Examining sacubitril/valsartan prescribing in this context therefore provides insight into geographic equity and the operational requirements for translating guideline recommendations into routine practice.

Implementation science offers a useful framework for this analysis. The updated Consolidated Framework for Implementation Research (CFIR), together with its outcomes addendum, organizes determinants across the intervention, outer setting, inner setting, individuals, implementation process, and implementation outcomes [33,34]. Proctor and colleagues distinguish adoption and fidelity from clinical effectiveness outcomes [35]. These concepts fit the present research question, which concerns prescribing practice and care-process completeness rather than re-testing clinical efficacy already established in randomized trials.

Accordingly, this study aimed to: (1) estimate active sacubitril/valsartan prescription and describe calendar-period variation during 2020–2025; (2) characterize selected physiological caution features and other documented contexts among patients without active therapy; (3) describe initiation, early laboratory monitoring, follow-up, dose escalation, current dose, highest documented dose, and reasons for current lowest-dose therapy; (4) identify the clearest remediable prescribing-process gaps without equating intermediate dosing with successful optimization; and (5) explain the quantitative patterns through clinician perspectives and integrated mixed-methods meta-inferences.

## 2. Materials and Methods

### 2.1. Study Design and Reporting

This study used an explanatory sequential mixed-methods design under a pragmatic paradigm [36,37,38]. The quantitative phase comprised a retrospective review of electronic health records (EHRs) to characterize sacubitril/valsartan adoption, the clinical context of non-prescription, initiation pathways, early monitoring, follow-up, dose reassessment, and titration. A preliminary quantitative review was completed before the qualitative phase and informed the interview guide and purposive sampling. The qualitative phase was then used to explain how clinicians interpreted the quantitative patterns and the organizational processes underlying them. Final integration occurred after the quantitative and qualitative datasets had been analyzed separately. The observational component was reported in accordance with STROBE, the qualitative component with the 32-item Consolidated Criteria for Reporting Qualitative Research (COREQ), and the integrated mixed-methods report with established mixed-methods reporting principles [39,40,41]. The completed COREQ checklist, semi-structured interview guide, and qualitative coding tree are provided as Supplementary Files S1, S2, and S3, respectively.

The study focused on prescribing practice and implementation processes rather than re-estimating the clinical efficacy of sacubitril/valsartan. Hospitalization, mortality, ventricular remodeling, and other treatment-effectiveness outcomes were therefore outside the prespecified scope.

### 2.2. Setting and Observation Period

The study was conducted at Al-Qurayyat General Hospital, a Ministry of Health regional public referral hospital serving Al-Qurayyat city and surrounding rural and border communities in northern Saudi Arabia. The hospital provides emergency and inpatient medicine, critical care, cardiology consultation, echocardiography, outpatient cardiology, clinical pharmacy, and hospital pharmacy dispensing. Sacubitril/valsartan was available on the institutional formulary in all three marketed strengths, although temporary dose-strength shortages could occur.

The quantitative observation window extended from 1 January 2020 through 31 December 2025. A preliminary quantitative review was undertaken before qualitative recruitment to identify patterns requiring explanatory exploration; qualitative interviews were conducted between November and December 2025. The final quantitative dataset was locked after completion of the observation window and was used for the final integrated analysis.

### 2.3. Quantitative Component

#### 2.3.1. Study Population and Sampling

Adults aged 18 years or older were eligible if the EHR contained a diagnosis of heart failure, a documented left-ventricular ejection fraction (LVEF) ≤ 40%, and at least one cardiology, internal medicine, inpatient, or heart-failure-related encounter during the observation period. Records were excluded when the HFrEF phenotype could not be confirmed, no eligible encounter was available, or medication and physiological information were materially incomplete. A census approach was used: all records meeting the eligibility criteria during the defined study period were included, and no sampling of eligible patient records was undertaken. Because the objective was to characterize the complete eligible service cohort, an a priori sample-size calculation was not used for the quantitative component. For this study, a heart-failure-related encounter was defined as an encounter in which HF/HFrEF was documented as an active clinical problem requiring evaluation or management; this could occur in cardiology, internal medicine, or inpatient care.

#### 2.3.2. Data Sources, Extraction, and Quality Assurance

Data were abstracted from the hospital EHR using a structured extraction form and data dictionary that prespecified variable definitions, time windows, dose equivalence, and rules for resolving conflicting documentation. Extracted variables included age, sex, nationality, body mass index, smoking status, LVEF, heart-failure duration and etiology, New York Heart Association functional class when documented, diabetes, hypertension, coronary artery disease, chronic kidney disease, atrial fibrillation, systolic blood pressure (SBP), serum creatinine, estimated glomerular filtration rate (eGFR), serum potassium, concomitant beta-blocker, mineralocorticoid receptor antagonist (MRA), and sodium–glucose cotransporter-2 (SGLT2) inhibitor therapy, outpatient-visit frequency, prescriber/cardiology involvement, initiation setting, pharmacist review, laboratory monitoring, follow-up, titration planning, appointment attendance, medication-supply problems, telemedicine contact, and dose history.

Heart-failure duration was abstracted when explicitly documented; however, exact onset dates were incomplete and could predate the institutional record, so duration was not analyzed as a continuous exposure. We therefore report the EHR-based distinction between newly diagnosed HFrEF (first documented in the available institutional record during the observation window) and established HFrEF rather than an exact duration measure.

R.O.A. performed the primary record abstraction. A second investigator independently re-extracted a random 10% sample of the quantitative cohort and all non-prescription cases; discrepancies were resolved by joint review of the source record and application of the prespecified data dictionary. Missingness was assessed for each variable. No statistical imputation was performed; descriptive denominators reflected available data, and variables with substantial missingness were interpreted cautiously. Dose-practice analyses were restricted to treated patients with at least six months of observable follow-up to reduce misclassification caused by insufficient opportunity for reassessment.

#### 2.3.3. Temporal Anchors and Prescribing-Process Measures

Distinct temporal anchors were defined to separate current treatment status, initiation, and subsequent follow-up. The current-status assessment date was the latest eligible encounter at which active medication status, SBP, potassium, serum creatinine, and eGFR could be aligned. The ARNI initiation date was the first documented sacubitril/valsartan prescription among treated patients. For time-to-prescription analyses, the ARNI consideration date was defined as the first HFrEF-related encounter after LVEF ≤ 40% had been documented at which medication status and contemporaneous SBP, potassium, and creatinine/eGFR data were available to assess ARNI use. Absence of the selected physiological caution features was not required for this definition. The prescribing interval was calculated from the ARNI consideration date to the first sacubitril/valsartan prescription. Each patient contributed to one mutually exclusive index-year cohort; consequently, year-stratified proportions describe calendar-period variation between patient cohorts rather than repeated annual prevalence estimates.

The primary quantitative outcome was active sacubitril/valsartan therapy at the current-status assessment, defined by an unexpired medication order or explicit documentation that the patient was taking the medicine. Historical prescriptions without evidence of continued use were not classified as active therapy. To characterize the clinical context of non-prescription, three measurable physiological caution features were examined: SBP < 100 mmHg, eGFR < 30 mL/min/1.73 m^2^, and potassium ≥ 5.5 mmol/L. These were treated as caution features rather than absolute contraindications or a complete eligibility algorithm. Additional documented contexts included ongoing ACE-inhibitor or angiotensin receptor blocker (ARB) therapy, previous renin–angiotensin-system intolerance, patient reluctance/refusal, and planned reassessment.

Among treated patients, initiation measures included inpatient/immediate post-discharge versus ambulatory initiation, switch from an ACE inhibitor/ARB versus ARNI initiation without documented prior ACE inhibitor/ARB exposure in the available EHR, cardiology-led versus other initiation, and residence outside the immediate hospital catchment. Among patients with at least six months of observable follow-up, post-initiation process measures included renal-function and potassium monitoring ordered and completed within 14 days, follow-up within six weeks, pharmacist medication review within 90 days, an explicitly named titration plan or responsible clinician, missed scheduled appointments, temporary dose-strength shortage, telemedicine contact, any dose-escalation attempt, escalation within 90 days, time to first escalation, starting dose, current dose, highest documented dose, and documented reasons for current lowest-dose therapy. The 14-day laboratory window reflects recommendations to reassess kidney function and potassium within approximately 1–2 weeks after initiation or dose increase [9]; the six-week and 90-day windows were pragmatic early-care benchmarks used to characterize continuity of reassessment.

Dose status was reported as 24/26 mg twice daily (lowest dose), 49/51 mg twice daily (intermediate dose), or 97/103 mg twice daily (target dose). Intermediate dosing was not automatically classified as successful optimization because the retrospective record did not consistently distinguish ongoing titration from stable clinically limited therapy or unresolved sub-target treatment. Among patients currently receiving 24/26 mg twice daily, reasons for remaining on or returning to the lowest dose were categorized as: (1) clinical/physiological limitation; (2) health-system/access limitation; (3) patient preference/adherence-related reason; or (4) no documented reason. Dose history was analyzed separately as never documented above 24/26 mg twice daily versus current lowest-dose therapy after a previously documented higher dose. Access limitations and patient preference/adherence were not considered pharmacological maximum tolerated dose. Exact starting-dose appropriateness and compliance with the 36-h ACE-inhibitor washout were not quantitatively adjudicated because the prior ACE inhibitor/ARB dose and precise timing of the final ACE-inhibitor administration were not consistently available.

The study did not define a binary “therapeutically optimized” endpoint or formally adjudicate a maximum tolerated dose for every patient. Target dose was reported separately, and sub-target therapy was interpreted using dose history, documented clinical/physiological limitations, access constraints, patient preference/adherence, and evidence of reassessment. Failure to reach 97/103 mg twice daily, or absence of a documented reason for non-escalation, was not by itself classified as inappropriate management or confirmed therapeutic inertia.

### 2.4. Qualitative Component

#### 2.4.1. Sampling and Recruitment

Clinicians involved in HFrEF medication decisions were recruited purposively to achieve variation in profession, seniority, and inpatient versus ambulatory responsibilities. Eligible participants included cardiologists, internists, clinical pharmacists, and cardiology/heart-failure nurses who contributed to ARNI initiation, monitoring, medication reconciliation, counseling, follow-up, or dose reassessment. Eighteen clinicians were approached directly by the qualitative researcher; 15 participated, and 3 did not participate. Reasons for non-participation were not recorded. Participation was voluntary, and managers were not informed of individual responses. Recruitment continued until analytic sufficiency was reached, operationalized as the point at which additional interviews no longer added materially new explanations of the principal quantitative findings, rather than by a prespecified numerical saturation threshold.

#### 2.4.2. Researcher Characteristics, Reflexivity, and Relationships

All interviews were conducted by Reham Olayan Alonayzan (R.O.A.), the first author, a female MSc student in Clinical Pharmacy and a researcher. Before data collection, she reviewed the interview guide and interviewing procedures with senior investigators and pilot-tested the guide for clarity, sequencing, and relevance. No additional formal qualitative-interviewing credential is claimed. R.O.A. had no supervisory, line-management, or performance-appraisal relationship with participants. No research relationship was established before recruitment; incidental professional familiarity within the hospital setting was possible but was not systematically recorded. Participants were informed of the study purpose, the interviewer’s clinical-pharmacy background, and that she was acting in a research rather than evaluative role.

The research team recognized that familiarity with HFrEF pharmacotherapy could facilitate clinically informed probing while also creating assumptions about what constituted appropriate prescribing. Reflexive memoing was therefore used throughout data collection and analysis to document assumptions, emerging interpretations, and decisions requiring discussion with the wider multidisciplinary research team.

#### 2.4.3. Interview Guide and Data Collection

A semi-structured interview guide was developed from the preliminary quantitative patterns, the study objectives, and CFIR-informed implementation domains. The guide explored: local normalization of sacubitril/valsartan; evidence strength and perceived relative priority; clinical suitability and safety judgment; blood-pressure, renal-function, and potassium considerations; prior ACE-inhibitor/ARB exposure and washout; starting-dose selection; laboratory monitoring and result review; titration responsibility; reasons for persistent or recurrent low-dose therapy; missed appointments and geographic access; pharmacy supply; patient understanding and adherence; documentation; multidisciplinary roles; and potential service improvements. The guide was pilot-tested before formal data collection, and its domains and core prompts are provided in Supplementary File S2.

Interviews were conducted face-to-face in English in a private hospital room, with no non-participant present. Interviews lasted approximately 28–46 min and were audio-recorded after written informed consent. No repeat interviews were conducted. Audio recordings were transcribed verbatim and de-identified; R.O.A. checked each transcript against the corresponding recording for accuracy. Contextual and reflexive notes were documented during and immediately after interviews to capture issues relevant to interpretation and subsequent probing. Full transcripts were not routinely returned to participants, and formal member checking of the final thematic structure was not undertaken; participants could clarify or expand their statements during the interview.

### 2.5. Data Analysis

#### 2.5.1. Quantitative Data Analysis

Quantitative data were analyzed using IBM SPSS Statistics, Version 28.0 (IBM Corp., Armonk, NY, USA). Continuous variables were summarized as mean ± standard deviation or median and interquartile range (IQR), according to distribution, and categorical variables as frequencies and percentages. Unadjusted comparisons between active-ARNI users and non-users used Welch’s independent-samples t test or the Mann–Whitney U test for continuous variables and the chi-square test or Fisher’s exact test for categorical variables, as appropriate. The active-prescription proportion was reported with a 95% confidence interval. Calendar-period variation was summarized descriptively across the mutually exclusive index-year cohorts; inferential trend testing was not used.

Multivariable explanatory or prediction models were not used as the primary inferential framework because several post-initiation care-process variables accrued after treatment initiation, could lie on the same implementation pathway as dose escalation, and could not be ordered uniformly relative to the outcome. In addition, the numbers of non-users and unresolved current lowest-dose cases were modest for stable multivariable modeling. Post-initiation findings were therefore interpreted as descriptive care-process patterns rather than independent causal effects. Two-sided *p*-values <0.05 were considered statistically significant for exploratory unadjusted comparisons, without multiplicity adjustment.

#### 2.5.2. Qualitative Data Analysis

Qualitative data were analyzed using the Framework Method with a hybrid deductive–inductive approach [33,42]. NVivo (Lumivero, Denver, CO, USA) was used for transcript management, coding, retrieval, memoing, and development of framework matrices. Analysis began with repeated familiarization with the transcripts and reflexive notes. Initial deductive codes were sensitized by CFIR domains and the quantitative findings that the qualitative phase was intended to explain, while inductive coding allowed unanticipated clinical and organizational issues to emerge from the interviews.

Two researchers independently coded three information-rich transcripts, compared coding decisions and interpretations, and developed an agreed working analytic framework. Coding differences were discussed to clarify code definitions and boundaries rather than treated as a statistical inter-rater reliability exercise. R.O.A. then coded the remaining transcripts in NVivo using the refined framework, with regular review by the second qualitative analyst and senior study team. Coded data were charted into participant-by-code matrices to support comparison across professional groups and inpatient versus ambulatory roles. Themes were developed iteratively by examining pattern, context, convergence, contradiction, and explanatory value. Negative and deviant cases were deliberately examined, including clinically vulnerable patients who were successfully titrated and apparently stable patients whose lowest-dose therapy persisted without a clear reassessment pathway. The final coding hierarchy and relationship between themes, subthemes, and key codes are presented in Supplementary File S3.

### 2.6. Qualitative Trustworthiness

Credibility was strengthened through transcript checking against audio recordings, analyst triangulation during initial coding, regular peer debriefing, integration with quantitative findings, and deliberate examination of negative/deviant cases. Dependability was supported by a documented analytic framework, NVivo coding records, reflexive memos, and an audit trail of code and theme refinement. Confirmability was supported by explicit reflexive consideration of the research team’s clinical-pharmacy assumptions and discussion of alternative interpretations. Transferability was facilitated through detailed description of the hospital context, participant professional groups, experience, and care-setting responsibilities. Because the study used an interpretive Framework Method approach, formal calculation of inter-coder agreement was not used as a measure of qualitative validity. Full transcript return and formal member checking of final themes were not performed and are reported transparently in the COREQ checklist (Supplementary File S1).

### 2.7. Mixed-Methods Integration

Integration occurred at the design, methods, analysis, and interpretation levels. The preliminary quantitative review informed the qualitative interview domains and focused probing on patterns requiring explanation. Purposive sampling ensured representation of clinicians occupying different positions in the medication pathway. After separate quantitative and qualitative analyses, findings were linked using joint displays and narrative weaving. Relationships between datasets were classified as convergence, complementarity, expansion, or discordance [36,37]. Meta-inferences were developed only when supported by both datasets or were explicitly identified as qualitative explanations of quantitative patterns. Final integration was summarized using a joint display and an integrated implementation pathway, which are presented in Section 3.10 (Mixed-Methods Integration).

### 2.8. Ethical Considerations

The study was approved by the Saudi Ministry of Health Institutional Review Board (Approval No. TU-077/025/352) and was conducted in accordance with the Declaration of Helsinki. The requirement for individual informed consent was waived for the retrospective EHR component because routinely collected data were analyzed in de-identified form. Written informed consent was obtained from all clinicians before the qualitative interviews and audio recording. Quantitative records and qualitative materials were de-identified and stored on password-protected, access-restricted devices available only to the research team.

### 2.9. Use of Generative Artificial Intelligence in Manuscript Preparation

During manuscript preparation, the authors used OpenAI ChatGPT (GPT-5.6 Sol; OpenAI, San Francisco, CA, USA) for language editing, organization, manuscript-development support, and consistency checking. The authors critically reviewed all AI-assisted output and take full responsibility for the manuscript. Generative AI was not used to collect participant data or make scientific decisions.

## 3. Results

### 3.1. Cohort Identification and Data Completeness

**Figure 1:**
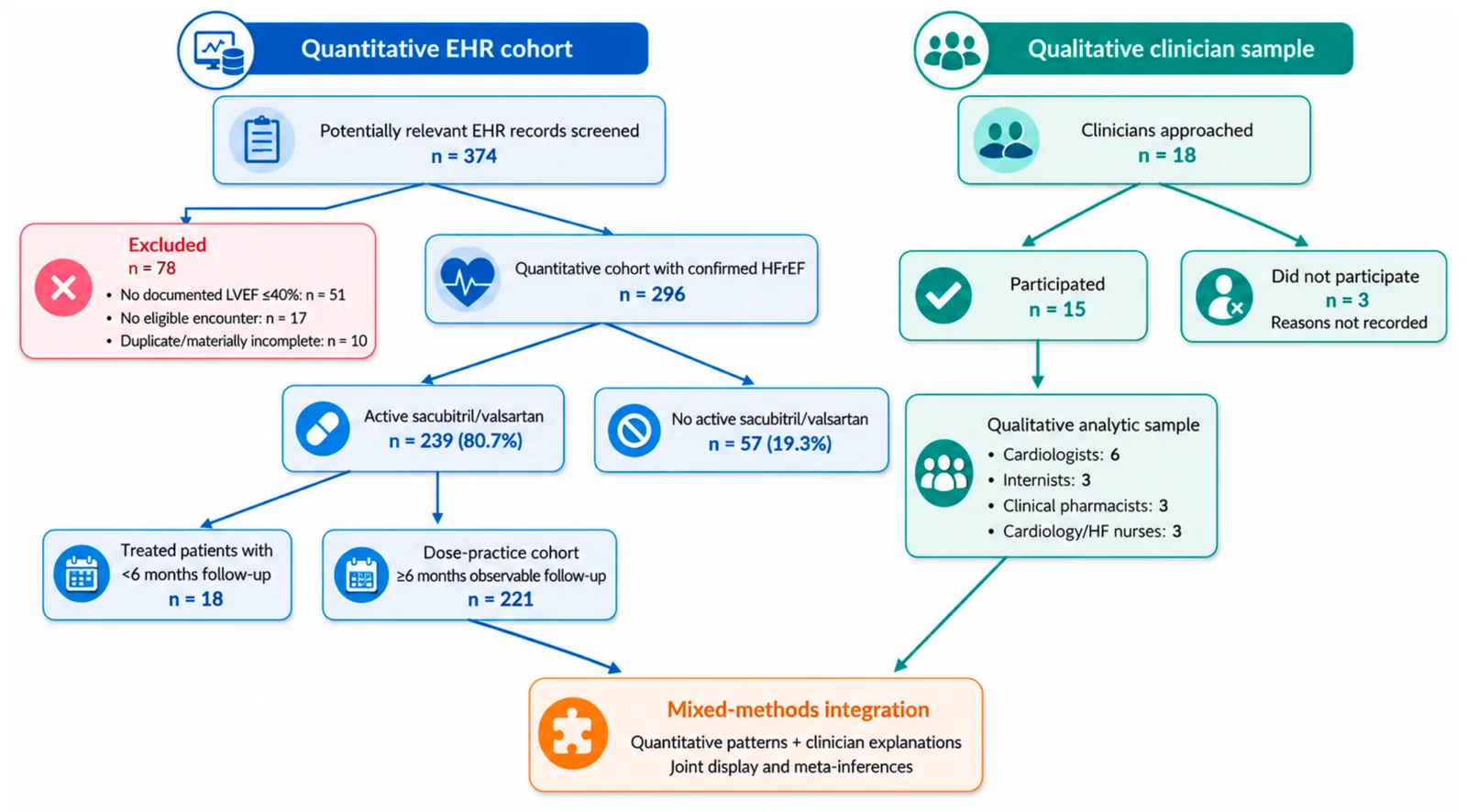
Study flow and analytic populations. Colors are used for visual distinction only: blue denotes the quantitative EHR pathway, green denotes the qualitative clinician pathway, red denotes exclusions, and orange denotes mixed-methods integration. Arrow colors correspond to their respective pathways and do not represent additional variables or analytical relationships.

Of 374 potentially relevant EHR records screened, 78 were excluded: 51 lacked a documented LVEF ≤40%, 17 had no eligible encounter during the observation period, and 10 were duplicates or materially incomplete. The quantitative cohort therefore comprised 296 adults with confirmed HFrEF. At the current-status assessment, 239 were receiving sacubitril/valsartan, and 57 were not, corresponding to an active-prescription proportion of 80.7% (95% CI 75.8–85.0%). Two hundred twenty-one treated patients had at least six months of observable follow-up and formed the dose-practice cohort. Eighteen clinicians were approached for the qualitative phase; 15 participated, and 3 did not participate, with reasons for non-participation not recorded (Figure 1).

### 3.2. Patient Characteristics and Unadjusted Associations

The mean age was 60.6 ± 12.5 years, 237 patients (80.1%) were men, and 264 (89.2%) were Saudi nationals. Mean LVEF was 31.2 ± 4.2%. Ischemic HF was documented in 195 patients (65.9%); diabetes in 190 (64.2%); hypertension in 219 (74.0%); coronary artery disease in 219 (74.0%); and chronic kidney disease in 76 (25.7%). Beta-blockers were prescribed to 280 (94.6%), MRAs to 192 (64.9%), and SGLT2 inhibitors to 145 (49.0%).

Demographic characteristics and LVEF were similar across prescription groups. Non-users had lower SBP, lower eGFR, and higher potassium, and chronic kidney disease was more common among non-users. MRA and SGLT2-inhibitor use were more common among patients with active ARNI therapy. These are concurrent cross-sectional patterns and should not be interpreted as independent determinants of prescribing (Table 1).

### 3.3. Calendar-Period Variation and Time to Prescription

**Figure 2:**
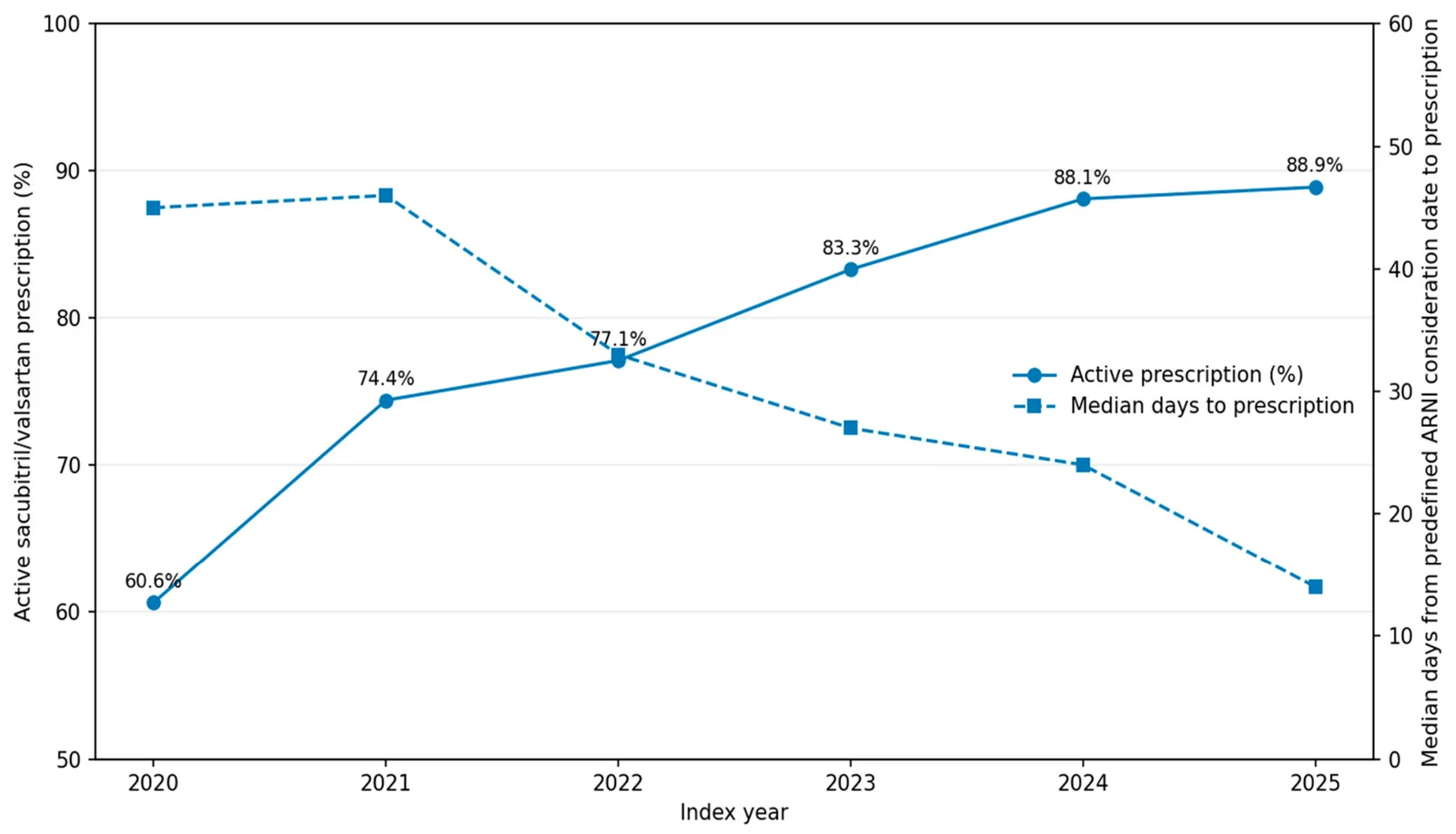
Calendar-period variation in active sacubitril/valsartan prescription and median documented time to prescription, 2020–2025. Each patient contributed to one index-year cohort.

Across mutually exclusive index-year cohorts, the proportion with active sacubitril/valsartan at the current-status assessment ranged from 60.6% among patients indexed in 2020 to 88.9% among those indexed in 2025. When grouped into two-year periods, the corresponding proportions were 68.1% in 2020–2021, 80.4% in 2022–2023, and 88.5% in 2024–2025. These values describe calendar-period differences between patient cohorts rather than repeated annual prevalence in the same population. Among treated patients, the median interval from the predefined ARNI consideration date to initial prescription was 26 days (IQR 12–46), declining from approximately 45 days in the earliest two-year period to 16.5 days in 2024–2025 (Table 2 and Figure 2).

### 3.4. Initiation Pathways

As shown in Table 3, newly diagnosed HFrEF accounted for 98 patients; adoption was 83.7% (82/98) in this group and 79.3% (157/198) among patients with established disease (*p* = 0.435). Among the 239 treated patients, 155 (64.9%) had documented prior ACE inhibitor or ARB exposure and were classified as switches, whereas 84 (35.1%) had no documented prior exposure and were categorized as ARNI initiation without documented prior ACE inhibitor/ARB exposure in the available EHR. Seventy-five (31.4%) were initiated during or immediately after hospitalization, 164 (68.6%) in ambulatory care, and 184 (77.0%) under a cardiology-led pathway. Eighty-eight treated patients (36.8%) lived outside the immediate hospital catchment area. This category describes the available record and does not establish that ARNI was deliberately selected as first-line therapy in every case; prior treatment outside the institutional record could not be excluded.

These findings indicate that sacubitril/valsartan use was not confined to newly diagnosed or hospitalized patients. Most initiation occurred in ambulatory care, and more than one third of treated patients lived outside the immediate catchment, increasing reliance on appointment continuity, laboratory access, and communication between visits.

### 3.5. Physiological Caution Features and Context of Non-Prescription

Among the 57 patients without active sacubitril/valsartan, 41 (71.9%) had at least one selected physiological caution feature at the status assessment: 32 had SBP < 100 mmHg, 16 had eGFR < 30 mL/min/1.73 m^2^, and 12 had potassium ≥ 5.5 mmol/L; 18 had at least two features. These findings describe measurable safety considerations rather than absolute contraindications. Among the full cohort, 197 patients had none of the three selected caution features, and 181 of these (91.9%) were receiving sacubitril/valsartan.

Sixteen non-users had none of the three selected physiological caution features. Fourteen of these 16 remained on an ACE inhibitor or ARB, indicating tolerance of some form of renin–angiotensin-system inhibition but not proving ARNI suitability. Across the entire non-user group, eight had previous intolerance documented, five had patient reluctance or refusal, and nine had a documented plan to reconsider therapy after stabilization or additional monitoring. Categories overlapped, and retrospective documentation did not permit definitive adjudication of appropriateness for every non-prescription decision. The selected caution features and other documented contexts among non-users are summarized in Table 4. Accordingly, this 16-patient subgroup was interpreted as warranting structured clinical review and explicit documentation rather than as a denominator of confirmed missed treatment opportunities.

### 3.6. Monitoring and Follow-Up After Initiation

Among 221 patients with at least six months of observable follow-up, a laboratory order for renal function and potassium within 14 days was documented for 180 (81.4%), and the test was completed within that interval for 166 (75.1%). Follow-up occurred within six weeks for 150 (67.9%), pharmacist review within 90 days for 139 (62.9%), and an explicitly named titration plan for 145 (65.6%). Fifty-two patients (23.5%) missed at least one scheduled appointment during the initial titration period, 26 (11.8%) had a documented temporary dose-strength supply problem, and 34 (15.4%) used telemedicine for at least one interim contact. Exact total ARNI exposure duration beyond the ≥6-month eligibility threshold was not consistently standardized in the source record and therefore was not summarized as a median or used for duration-stratified target-dose analyses. Accordingly, target-dose attainment in this cohort should be interpreted as treatment status at the latest eligible assessment among patients with at least six months of observation, not as a duration-standardized cumulative probability of reaching target dose.

**Figure 3:**
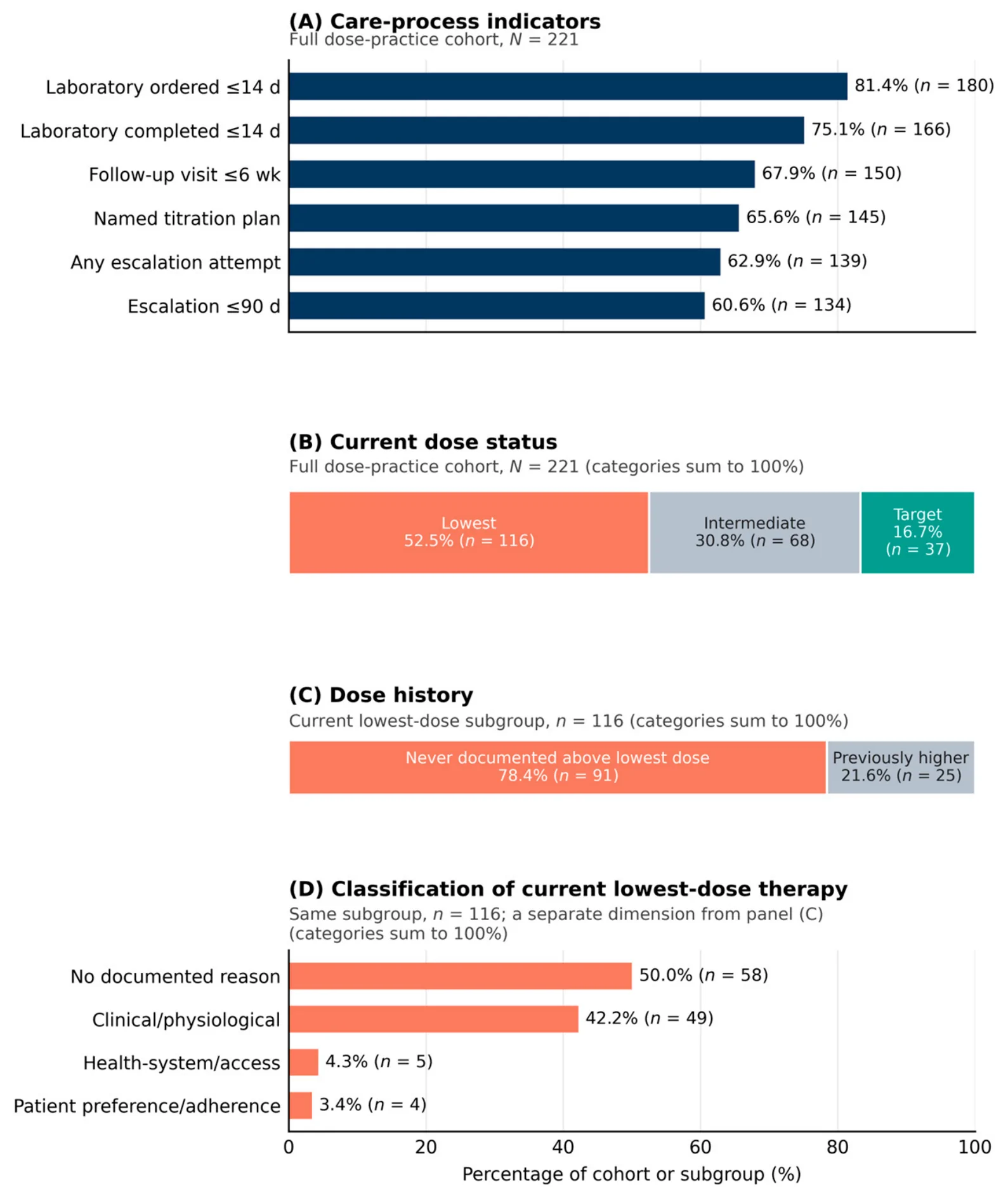
Post-initiation sacubitril/valsartan care-process indicators, current dose status, dose history, and classification of current lowest-dose therapy. (**A**) Care-process indicators among the full dose-practice cohort (*n* = 221), including laboratory monitoring, early follow-up, named titration planning, and dose-escalation attempts. (**B**) Current sacubitril/valsartan dose distribution in the same cohort. (**C**) Dose history among patients currently receiving the lowest dose (24/26 mg twice daily; *n* = 116), distinguishing those never documented above the lowest dose from those who had previously reached a higher dose. (**D**) Classification of current lowest-dose therapy in the same subgroup according to documented clinical/physiological limitation, health-system/access limitation, patient preference/adherence, or absence of a documented reason. Percentages in panels (**A**,**B**) use *n* = 221 as the denominator; percentages in panels (**C**,**D**) use *n* = 116. Dose-history and documented-reason categories represent separate dimensions.

The gaps between ordering laboratory tests, completing them, attending early follow-up, documenting responsibility, and attempting dose escalation illustrate attrition across the post-initiation pathway. These descriptive measures should not be interpreted as independent causal effects, because several occur sequentially and may share common organizational determinants (Table 5 and Figure 3).

### 3.7. Dose Selection, Escalation, and Current Sub-Target Therapy

At initiation, 166 patients (75.1%) received 24/26 mg twice daily and 55 (24.9%) received 49/51 mg twice daily. At the current-status assessment, 116 (52.5%) were receiving 24/26 mg twice daily, 68 (30.8%) were receiving 49/51 mg twice daily, and 37 (16.7%) were receiving the target dose of 97/103 mg twice daily. The highest documented dose differed from the current dose because of subsequent reductions: 48 patients (21.7%) had reached target dose at some point, 82 (37.1%) reached the intermediate dose, and 91 (41.2%) never exceeded the lowest dose.

An escalation attempt was documented in 139 patients (62.9%). Median time to first escalation was 29 days (IQR 19–44), and 134 patients (60.6% of the dose-practice cohort) had an escalation attempt within 90 days. Among patients who reached the target dose, the median documented time to target was 105.5 days (IQR 67.3–136.5). Dose-history reconciliation showed that, among the 116 patients currently receiving 24/26 mg twice daily, 91 had never been documented above that dose, whereas 25 had previously reached either 49/51 mg twice daily or 97/103 mg twice daily before later returning to 24/26 mg twice daily. Event-level EHR documentation did not consistently distinguish formal dose reduction, temporary interruption, or restart at a lower dose; therefore, a separate aggregate count of reduction/interruption events was not reported.

Of the 116 patients currently receiving 24/26 mg twice daily, 58 had a documented reason for remaining on or returning to the lowest dose, and 58 had no documented reason for non-escalation or continued low-dose therapy. The 58 documented reasons comprised clinical/physiological limitations in 49 patients (42.2% of the current lowest-dose subgroup), comprising symptomatic hypotension in 23/116 (19.8%), renal dysfunction in 12/116 (10.3%), hyperkalemia in 8/116 (6.9%), and frailty or orthostatic symptoms in 6/116 (5.2%), health-system or access limitations in 5 (4.3%), and patient preference or adherence concerns in 4 (3.4%). These categories were reported separately rather than combined into a single “optimized” endpoint. Access limitations and patient preference/adherence were not labeled as pharmacological maximum tolerated dose. The EHR did not consistently permit intermediate-dose patients to be separated into active titration, clinically limited maintenance, and unresolved sub-target therapy. Dose selection, dose history, escalation, and the classification of current lowest-dose therapy are summarized in Table 6. Absence of a documented reason was treated as documentation/reassessment uncertainty rather than as confirmed therapeutic inertia or proof of inappropriate prescribing.

### 3.8. Qualitative Participant Characteristics

Eighteen clinicians were approached, and 15 participated (six cardiologists, three internists, three clinical pharmacists, and three cardiology/HF nurses); three did not participate, and reasons were not recorded. Median professional experience among participants was 9 years (range 3–22), and 11 had worked at the hospital for at least five years. Nine contributed primarily to outpatient care, four to inpatient or critical care, and two across both settings. The sample represented the medication pathway from hospitalization and reconciliation through laboratory monitoring, counseling, and ambulatory titration. Participant characteristics are summarized in Table 7.

### 3.9. Qualitative Findings

Five themes explained how high active ARNI use coexisted with incomplete post-initiation process fidelity. Themes were observed across professional groups, although each profession emphasized different parts of the pathway. Negative cases—patients with substantial physiological vulnerability who were successfully titrated and apparently stable patients who remained at the starter dose without a clear reassessment pathway—were used to refine the interpretation.

#### 3.9.1. Sacubitril/Valsartan Had Become Normalized as Foundational Therapy

Participants consistently described sacubitril/valsartan as expected therapy rather than an exceptional escalation. Guideline endorsement, formulary access, and growing local experience reduced uncertainty about whether the medicine should be considered. Several clinicians contrasted current practice with the earlier period, when switching was delayed until repeated admissions or persistent symptoms.

*“At the beginning, we selected only the obvious patients. Now the question is usually why the patient is not on it, not whether we should think about it.”*

*(P03, cardiologist)*

Normalization was strongest among cardiologists and pharmacists, but internists also described greater confidence after local protocols and repeated exposure. The higher active use and shorter prescribing delays observed in later index-year cohorts were therefore consistent with growing institutional familiarity, although the calendar analysis cannot establish causation.

#### 3.9.2. Clinical Suitability Was a Dynamic Safety Judgment, Not a Threshold Checklist

Blood pressure, kidney function, and potassium were central, but clinicians rarely interpreted one measurement in isolation. They considered symptoms, trajectory, diuretic burden, congestion, recent acute kidney injury, and whether monitoring could be repeated. The same SBP could lead to different decisions in an asymptomatic stable patient and a patient with dizziness or recent falls.

*“A pressure of ninety-eight is not one story. If the patient is walking, not dizzy, and I can recheck next week, I may proceed. If the patient lives far away and I will not see the laboratory result, I become more conservative.”*

*(P06, cardiologist)*

A negative case involved a patient with moderate chronic kidney disease who was successfully titrated through small steps and scheduled laboratory review. This case challenged a static interpretation of reduced eGFR as treatment exclusion and supported the view that physiological measurements require clinical context and monitoring capacity.

#### 3.9.3. Monitoring Was the Hidden Work of Prescribing

Clinicians distinguished the act of writing the initial prescription from the work required to maintain and escalate it. Laboratory ordering was usually straightforward; ensuring that testing occurred, reviewing the result promptly, contacting the patient, and authorizing the next dose were less reliable. Participants described each step as a possible point of failure.

*“The order is easy. The difficult part is who checks the creatinine, who calls the patient, and who changes the dose before the next routine appointment.”*

*(P08, clinical pharmacist)*

This theme helped explain the observed attrition between laboratory ordering, laboratory completion, early follow-up, and dose escalation. Participants emphasized that a completed laboratory test did not necessarily lead to action unless a named clinician reviewed the result and reopened the dose decision.

#### 3.9.4. Current Low-Dose Therapy Had Multiple Meanings

Participants strongly rejected equating every low dose with poor care. They described several distinct reasons for current lowest-dose therapy. In some patients, 24/26 mg twice daily represented a clinically limited dose after symptomatic hypotension, renal dysfunction, hyperkalemia, frailty, or orthostatic symptoms. In others, health-system/access constraints or patient preference/adherence concerns limited escalation without constituting pharmacological intolerance. Clinicians also distinguished patients who had never progressed above the starter dose from those whose dose had previously been increased and later returned to 24/26 mg twice daily. A further group simply remained on the initial dose because no reassessment had been scheduled or responsibility was diffuse. Participants therefore regarded dose history, the documented reason, and the planned reassessment as central to interpreting sub-target therapy.

*“There is a big difference between ‘we tried and this is the tolerated dose’ and ‘it stayed like this because nobody reopened the decision.’ The record often does not show that difference.”*

*(P12, cardiologist)*

The apparently stable but unreviewed patient was the principal deviant case: clinicians agreed that such patients were easily overlooked because the medication list looked guideline concordant. Participants therefore viewed documentation of the reason for non-escalation as part of prescribing quality rather than an administrative detail.

#### 3.9.5. Multidisciplinary Ownership and Access Determined Whether Intention Became Action

Pharmacists and nurses were described as particularly valuable for medication reconciliation, ACE-inhibitor washout, counseling, laboratory review, appointment reminders, and escalation prompts. Clinicians did not view these roles as replacing physician judgment; they viewed them as preserving continuity between decisions. Geographic distance, missed visits, and intermittent dose availability added friction, especially when no rapid-access pathway existed.

*“When the plan says check potassium, call the patient, and increase if acceptable, the dose moves. When the plan only says ‘follow in clinic,’ it may stay unchanged for months.”*

*(P04, clinical pharmacist)*

Participants proposed a standardized initiation order set, automatic laboratory reminders, a registry of patients below target or undocumented maximum tolerated dose, pharmacist- or nurse-led interim contacts, and protected rapid-access appointments. They emphasized that audit should distinguish physiologically limited patients from those who had not been reassessed.

The five themes and their explanatory contributions are summarized in Table 8.

### 3.10. Mixed-Methods Integration

Integration produced five principal meta-inferences. First, high active use and shorter prescribing delays in later index-year cohorts were consistent with maturation of the local HFrEF treatment pathway, but the calendar analysis was not a repeated annual prevalence study. Second, selected physiological caution features were common among non-users, while clinician accounts showed that blood pressure, renal function, potassium, symptoms, trajectory, and monitoring capacity were interpreted dynamically. Third, post-initiation work was sequential: laboratory completion, result review, follow-up, communication, and a renewed prescribing decision were separate steps. Fourth, target-dose attainment was low, and intermediate dosing could not be assumed to represent successful optimization. Fifth, the clearest measurable residual gap was current lowest-dose therapy without a documented reason for non-escalation or reassessment plan.

**Figure 4:**
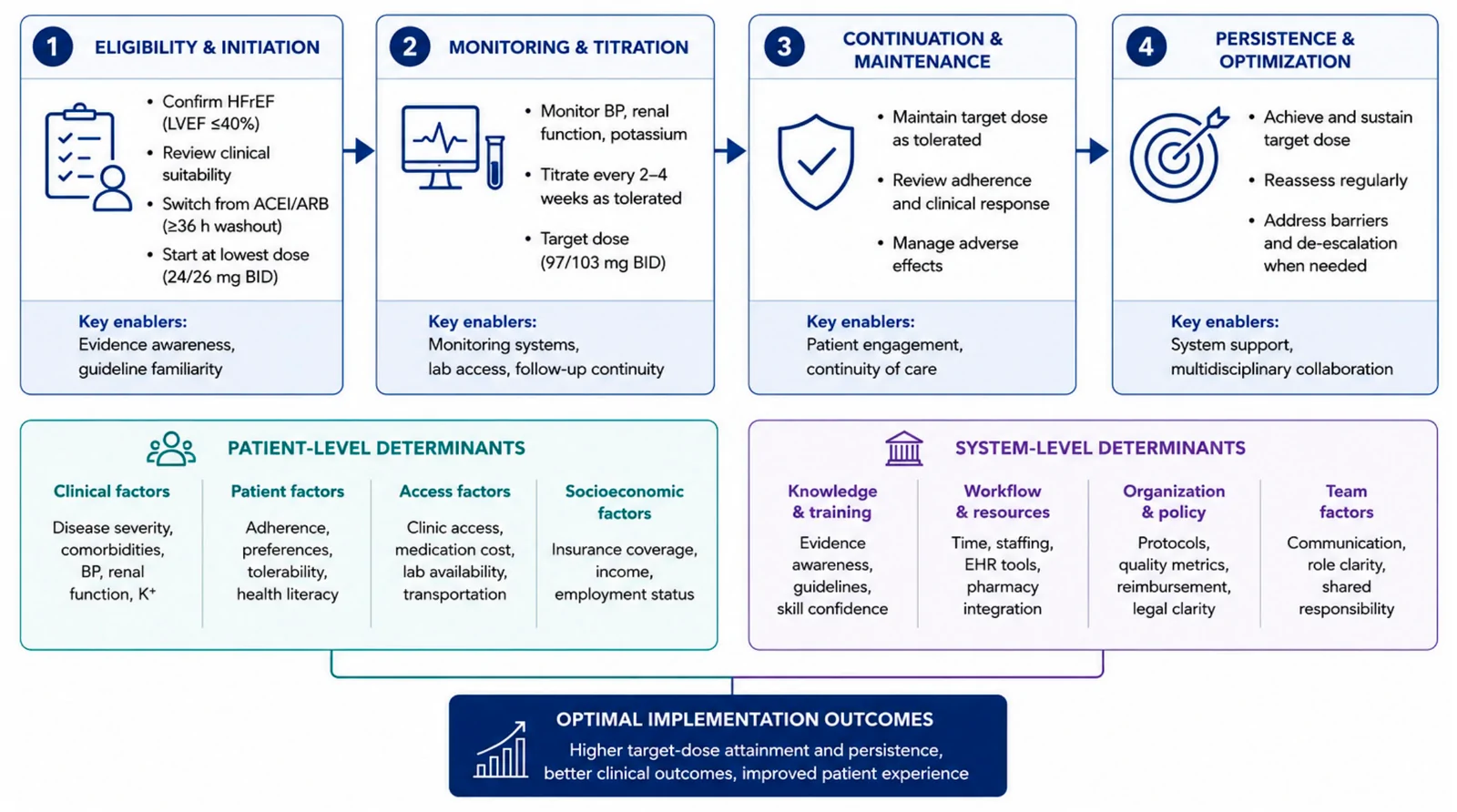
Sacubitril/valsartan implementation pathway in HFrEF care. The figure summarizes the sequential stages from eligibility and initiation through monitoring, titration, maintenance, and dose optimization, highlighting key patient- and system-level determinants that may influence implementation. Arrows indicate pathway relationships rather than proven causality.

No major discordance was identified between datasets, but the qualitative findings expanded the meaning of several descriptive patterns. Geographic distance, for example, was described as important mainly when it disrupted laboratory testing, appointment continuity, and communication. Similarly, pharmacists and nurses were described as continuity mechanisms within the pathway, but the retrospective quantitative design does not establish an independent causal effect of pharmacist review. The integrated quantitative and qualitative findings are summarized in Table 9 and synthesized visually as the implementation pathway shown in Figure 4.

## 4. Discussion

### 4.1. Principal Findings

This explanatory sequential mixed-methods study provides a detailed account of sacubitril/valsartan prescribing practice in a northern Saudi HFrEF service. Four findings are central. First, active therapy was high at 80.7%, and patients indexed in later calendar periods had higher active ARNI use and shorter documented prescribing delays, although the calendar cohorts are descriptive rather than repeated annual prevalence estimates. Second, 71.9% of non-users had at least one selected physiological caution feature, although these features were not equivalent to absolute contraindications. Third, the larger residual gap occurred after initiation: only 16.7% were currently receiving 97/103 mg twice daily and 21.7% had reached target at any point. Fourth, among 116 patients currently receiving 24/26 mg twice daily, 91 had never been documented above the lowest dose, and 25 had previously reached a higher dose before returning to it; 49 had a clinical/physiological reason, 5 a health-system/access reason, 4 a patient preference/adherence reason, and 58 had no documented reason for current low-dose therapy. Clinician interviews described monitoring continuity, explicit responsibility, appointment access, and communication as central to whether the dose decision was revisited.

The study therefore reframes ARNI implementation away from both a single prescribing indicator and a single dose-completion indicator. Active prescription can conceal incomplete follow-up, whereas target-dose attainment alone can misclassify patients who are genuinely clinically limited. At the same time, intermediate dosing should not automatically be labeled “optimized” because it may reflect active titration, a documented sub-target ceiling, or unresolved sub-target persistence. A more defensible quality framework reports these states separately.

### 4.2. Adoption in International and Saudi Context

The 80.7% active-prescription proportion is higher than many early real-world reports. CHAMP-HF and other registries documented substantial underuse of ARNI and other foundational therapies even in cardiology settings [18,19]. More recent real-world programs demonstrate substantially greater uptake as evidence, formulary access, and specialist pathways mature [22,27,32,43]. The present study differs from treatment-only cohorts by including both users and non-users and by explicitly mapping the post-initiation care process.

The calendar pattern in Al-Qurayyat is informative but should be interpreted cautiously. The year-specific groups are mutually exclusive index-year cohorts rather than repeated cross-sections of the same denominator. Nonetheless, later cohorts had both a higher proportion with active ARNI therapy and shorter documented intervals to prescription. Together with clinician accounts of increasing familiarity, this pattern is consistent with temporal maturation of the local HFrEF treatment pathway rather than proof of a specific institutional learning effect.

Saudi studies have shown growing use and favorable real-world experience with sacubitril/valsartan [28,29,30], but most have focused on outcomes among treated patients. The present implementation focus complements that literature by examining non-prescription context, monitoring completion, dose reassessment, and documentation quality within a defined HFrEF service. This pathway-level perspective is also distinct from recent JCM real-world studies that primarily examined longitudinal treatment introduction or clinical response [32,43].

Although this study was designed around ARNI implementation, the medication profile also indicates broader opportunities for comprehensive guideline-directed medical therapy (GDMT): beta-blocker use was 94.6%, whereas MRA and SGLT2-inhibitor use were 64.9% and 49.0%, respectively; among active ARNI users, SGLT2-inhibitor use was 53.6%. The 2020–2025 observation period spans the rapid incorporation of SGLT2 inhibitors into foundational HFrEF therapy [6,7,8,9,15,16,17], but the present cross-sectional medication snapshot cannot determine whether non-use reflected calendar-time adoption, contraindication or intolerance, prior adverse effects, patient preference, or other clinical or health-system factors. Concomitant doses and complete class-specific contraindication data for the other GDMT classes were not sufficiently complete to adjudicate their optimization. Accordingly, the ARNI findings should be interpreted within the broader goal of implementing all four foundational therapies rather than as a stand-alone measure of total GDMT quality.

### 4.3. Clinical Context of Non-Prescription

Non-prescription should not automatically be labeled potential therapeutic inertia. Approximately 72% of non-users had at least one selected physiological caution feature. However, these variables should not be treated as a rigid eligibility algorithm. Contemporary ACC guidance lists SBP < 100 mmHg as a caution and recommends a reduced starting dose of 24/26 mg twice daily in severe kidney impairment rather than excluding sacubitril/valsartan solely because eGFR is <30 mL/min/1.73 m^2^ [9]. Renal function and potassium therefore require clinical interpretation, trajectory assessment, and monitoring rather than threshold-only decision-making [9,44].

The qualitative findings added necessary nuance. Clinicians interpreted safety dynamically, considering symptoms, trends, diuretic burden, recent instability, and access to reassessment. This is clinically appropriate: a single value does not capture the distinction between stable chronic kidney disease and evolving acute kidney injury, or between asymptomatic low-normal pressure and symptomatic hypotension. The results should therefore not be used to impose rigid thresholds.

Nevertheless, 16 non-users lacked the three selected caution features, and 14 remained on an ACE inhibitor or ARB. This subgroup is a reasonable target for structured review, not because ARNI was proven appropriate in every case, but because tolerance of another renin–angiotensin-system agent and absence of the selected measurable cautions warrant explicit documentation of the decision. Retrospective EHR review cannot establish appropriateness without formal clinical adjudication, and the present manuscript therefore describes context rather than labeling individual decisions correct or incorrect.

### 4.4. Initiation Pathways and the Importance of Time

Almost two-thirds of treated patients were switched from an ACE inhibitor or ARB, while one-third had no documented prior ACE inhibitor/ARB exposure in the available EHR. Inpatient or immediate post-discharge initiation accounted for 31.4%, consistent with evidence that stabilized hospitalized patients can begin sacubitril/valsartan before discharge or early afterward [11,12]. Most initiation nevertheless occurred in ambulatory care, placing greater weight on laboratory access, follow-up capacity, and medication counseling. An important limitation of the retrospective record was that exact prior ACE inhibitor/ARB dose and the precise interval after the last ACE-inhibitor administration were not consistently available; consequently, this study does not claim quantitative compliance with the 36-h washout requirement or formal starting-dose appropriateness. These should be incorporated into future prospective audit tools. This should not be interpreted as proof of intentional first-line ARNI use in every case: the retrospective record did not capture a reliable patient-specific reason for this pathway and could miss treatment received outside the institution.

The documented reduction in time to prescription across later index-year cohorts is a potentially useful prescribing-process signal. Delayed implementation leaves patients on incomplete guideline-directed therapy despite known benefit, and contemporary pathways emphasize rapid initiation and titration of foundational HFrEF treatment [9,25,45]. Because the present calendar groups were mutually exclusive cohorts, however, the finding should be interpreted as calendar-period variation rather than a formal interrupted time-series or repeated-prevalence analysis.

### 4.5. The Post-Initiation Gap: Target Dose, Clinical Limitation, and Other Reasons for Sub-Target Therapy

Target-dose attainment was low. Only 16.7% were currently receiving 97/103 mg twice daily, and 21.7% had reached that dose at any point. This contrasts with the REAL.IT pharmacoutilization analysis, in which 45.2% of patients with available final-dose data reached 97/103 mg twice daily, with mean titration to target of approximately seven weeks [27]. Cross-study comparisons must be cautious because patient selection, follow-up duration, health-system organization, and documentation differ, but the contrast reinforces that high drug uptake does not guarantee rapid target-dose attainment. Because ARNI exposure duration beyond the ≥6-month eligibility threshold was heterogeneous and not standardized, the 16.7% current target-dose proportion represents status at the latest eligible assessment rather than a duration-standardized probability of target-dose attainment.

The revised analysis deliberately avoids a composite endpoint that automatically counts 49/51 mg twice daily as successful optimization. Intermediate dosing is clinically ambiguous without knowing whether the patient is still being titrated, has a documented sub-target ceiling, or has simply not been reassessed. The current lowest-dose subgroup was therefore examined in two complementary ways. First, dose history showed that 91/116 had never been documented above 24/26 mg twice daily, whereas 25 had previously reached a higher dose and later returned to the lowest dose. Second, the reason for current lowest-dose therapy was clinical/physiological in 49, health-system/access-related in 5, patient preference/adherence-related in 4, and undocumented in 58. This dual classification is more actionable than a simple “target versus non-target” comparison because it distinguishes failure to progress from de-escalation after prior titration and separates pharmacological tolerance from system barriers and patient-level preferences. Likewise, the study does not claim that every sub-target dose represents a formally established maximum tolerated dose; that designation requires explicit clinical evidence of a ceiling to further escalation.

This distinction has implications for quality measurement. A target-dose-only indicator can penalize appropriate caution in frail, hypotensive, or renally vulnerable patients. Conversely, an “any ARNI prescription” indicator can conceal prolonged sub-target therapy without reassessment. A stronger audit framework records the current dose, highest attempted dose, date of last dose review, documented reason for non-escalation, and next review plan. Intermediate-dose patients should then be classified as active titration, documented clinically limited therapy, or unresolved sub-target therapy whenever the record permits.

### 4.6. Monitoring, Follow-Up, and Multidisciplinary Ownership

The quantitative process profile showed progressive attrition after initiation: 81.4% had laboratory monitoring ordered within 14 days, 75.1% completed it, 67.9% attended follow-up within six weeks, 65.6% had a named titration plan, and 60.6% had an escalation attempt within 90 days. These figures should be interpreted as sequential care-process completion rather than independent causal effects. The qualitative findings explained why: ordering a test, completing it, reviewing the result, contacting the patient, and authorizing the next dose were distinct operational steps.

Pharmacist review occurred in 62.9% of the dose-practice cohort. The retrospective design does not establish that pharmacist review independently caused better titration, because review may also mark a more organized care pathway. Nevertheless, systematic reviews and implementation studies support pharmacist-involved HF care as a means of strengthening medication reconciliation, monitoring, titration, and adherence [46,47,48,49,50], and the clinician interviews identified plausible local roles including washout verification, counseling, laboratory review, and escalation prompts.

Named responsibility emerged as equally important. A plan that states only “follow in clinic” does not identify who reviews laboratory values or when the dose decision should be reopened. Standardized order sets and collaborative practice protocols can assign specific actions to cardiologists, pharmacists, nurses, and patients without transferring ultimate clinical accountability. This approach is consistent with recent expert pathways emphasizing rapid implementation and structured reassessment [9,25,26].

### 4.7. Geographic and Organizational Context

More than one third of treated patients lived outside the immediate catchment, 23.5% missed at least one scheduled appointment during the initial titration period, and 11.8% experienced a documented temporary dose-strength supply problem. These variables were not modeled as independent causal predictors because they can interact with the same care pathway. Qualitative accounts nevertheless showed how geographic and organizational friction could accumulate through delayed laboratory testing, missed visits, and communication gaps.

Mechanistically, these constraints can interrupt the sequence required for safe escalation: delayed or missed laboratory testing, delayed result review, missed or late appointments, travel burden, temporary dose-strength shortage, and diffuse responsibility can postpone the next dose decision even when escalation may remain clinically feasible. The qualitative data therefore provide a plausible explanation for how access and organizational friction can delay reassessment, rather than an independently estimated causal effect.

Temporary dose-strength shortages affected a minority but were described as disruptive when they occurred during a narrow titration window. The broader implication is that isolated resource measures are less informative than whether the system can recover from a disruption. Rapid substitution, remote contact, laboratory review, and clear responsibility may prevent a short interruption from becoming prolonged sub-target therapy.

### 4.8. Implications for Heart Failure Management and Multidisciplinary Care

The findings suggest that improving sacubitril/valsartan implementation requires attention to the entire prescribing pathway rather than focusing only on whether ARNI therapy has been prescribed or whether the target dose has been reached. At the adoption stage, HFrEF services should systematically identify patients who are not receiving ARNI therapy and ensure that the clinical rationale for continued ACE inhibitor/ARB therapy or non-initiation is documented. At initiation, prior renin–angiotensin-system inhibitor exposure and, when relevant, the exact timing of ACE-inhibitor discontinuation should be recorded to permit prospective assessment of starting-dose selection and compliance with the recommended 36-h washout period. Each initiation or dose increase should also be linked to planned renal-function and potassium reassessment within approximately 1–2 weeks, an early follow-up encounter, and explicit assignment of responsibility for reviewing laboratory results and making the subsequent dose decision.

The post-initiation pathway should similarly be evaluated as a longitudinal process rather than by target-dose attainment alone. Clinical audit should distinguish the current dose from the highest dose previously reached and identify whether patients receiving sub-target therapy are undergoing active titration, have a documented clinical or physiological limitation, face a health-system or access barrier, have a patient preference or adherence-related reason, or have no documented explanation for non-escalation. This distinction is particularly important for patients currently receiving 24/26 mg twice daily because current lowest-dose treatment can represent very different clinical trajectories, including patients who have never been titrated above the starting dose and patients who previously reached a higher dose but subsequently required de-escalation. Accordingly, the most informative quality indicators are not simply the proportions prescribed ARNI or receiving the target dose, but also the time from documented ARNI consideration to initiation, completion of early laboratory monitoring, timely clinical reassessment, documentation of titration responsibility, dose-reassessment attempts, dose history, reasons for sub-target therapy, and the presence of a defined next review plan.

These processes are well suited to a multidisciplinary model of care. Cardiologists and other prescribing clinicians retain responsibility for clinical decision-making, while clinical pharmacists and heart-failure nurses can support medication reconciliation, verification of ACE-inhibitor washout, patient counseling, laboratory-result review, appointment coordination, and prompts for dose reassessment. In geographically peripheral services such as the setting examined in this study, a relatively simple infrastructure—including a shared EHR registry of patients below target dose, automated laboratory reminders, named responsibility for follow-up, pharmacist- or nurse-supported interim contact, and protected rapid-access appointments—may improve continuity without requiring a stand-alone specialist HF center. Telemedicine may further support this pathway, although its effectiveness depends on timely access to laboratory testing and the ability of the clinical team to act promptly on the results. Where feasible, these functions can be organized through structured heart-failure clinics or protocolized multidisciplinary pathways involving specialist nurses and clinical pharmacists. The specific organizational strategies proposed here were not prospectively evaluated in this study and should therefore be regarded as implementation hypotheses for future testing rather than established effects.

### 4.9. Strengths and Limitations

The study has several strengths. It examined a defined HFrEF service rather than only patients already receiving sacubitril/valsartan, and it separated current prescription, non-prescription context, initiation, monitoring, follow-up, dose escalation, current dose, highest dose, and documented reasons for current lowest-dose therapy. It also avoided assuming that every intermediate dose represents successful optimization. Finally, the explanatory sequential design linked quantitative care-process losses with clinician accounts and explicitly considered deviant cases.

The study was conducted at one regional public hospital and may not represent metropolitan tertiary centers, private hospitals, or facilities with different formularies, staffing, and follow-up models. Its value lies in detailed contextual analysis rather than national prevalence estimation. The index-year analysis used mutually exclusive cohorts rather than repeated annual cross-sectional denominators, so calendar differences should not be interpreted as a formal longitudinal prevalence trend.

The quantitative phase relied on retrospective EHR data. Active orders may not equal dispensing or ingestion, undocumented external prescriptions could be missed, and reasons for non-prescription or non-escalation may not have been recorded. “No documented reason for non-escalation” therefore indicates an information and process gap, not proof of inappropriate care.

The analysis used three selected physiological caution features rather than a complete treatment-eligibility algorithm. These measures did not capture every relevant consideration, including angioedema history, pregnancy, acute clinical instability, patient goals, symptom trajectory, or nuanced volume status. eGFR < 30 mL/min/1.73 m^2^ and SBP < 100 mmHg were treated as cautions requiring individualized decision-making, not automatic exclusions.

The dose-practice cohort excluded patients with less than six months of observable follow-up. This improved opportunity for longitudinal assessment but may have excluded recent initiations and altered calendar comparisons. Exact total duration from ARNI initiation to the current-status assessment was not consistently standardized for all patients, precluding a median exposure-duration estimate and duration-stratified target-dose analysis. Exact prior ACE inhibitor/ARB doses and the precise timing of the last ACE-inhibitor administration were also not consistently available, preventing robust quantitative adjudication of starting-dose appropriateness and 36-h washout compliance. Thus, comparisons of target-dose attainment across patients with different exposure durations remain vulnerable to unequal opportunity for titration. Exact heart-failure onset dates were also incompletely documented, which is why disease history is reported primarily as newly diagnosed versus established HFrEF rather than as a continuous duration measure.

Intermediate-dose patients could not be reliably classified as active titration, documented clinically limited therapy, or unresolved sub-target therapy from the retrospective record. For that reason, the final analysis did not use a composite “optimized prescribing” endpoint and did not retain multivariable models that could mix temporally overlapping post-initiation variables. Residual confounding and care-intensity bias remain relevant to all descriptive group comparisons.

Clinical outcomes were not evaluated; therefore, no inference can be made that incomplete dose reassessment, persistent sub-target dosing, or undocumented reassessment caused differences in heart-failure hospitalization, cardiovascular or all-cause mortality, or ventricular function.

The qualitative sample was limited to clinicians at the same institution. Patient perspectives on travel, adherence, twice-daily dosing, laboratory access, and preferences were represented through clinician accounts rather than direct interviews. The interviewer worked within a clinically related academic field, and incidental professional familiarity with some participants was possible; reflexive journaling was used to address this influence. Accordingly, adherence, transportation, treatment-preference, and access barriers identified qualitatively represent clinician interpretations rather than direct patient-reported experience. Future multisite work should incorporate patients and caregivers to test and extend these explanations.

## 5. Conclusions

Sacubitril/valsartan was widely used in this northern Saudi HFrEF service, and later mutually exclusive index-year cohorts descriptively showed higher active use with shorter documented prescribing delays; this pattern should not be interpreted as a longitudinal prevalence trend. The principal residual weakness occurred after initiation: laboratory completion, early follow-up, explicit titration ownership, and dose escalation were not consistently completed as one coordinated pathway. Only 16.7% were currently at target dose, but this percentage should not be interpreted as evidence that all sub-target dosing was inappropriate. Among 116 patients currently receiving the lowest dose, 91 had never been documented above it, and 25 had previously reached a higher dose before returning to 24/26 mg twice daily; reasons for current lowest-dose therapy were clinical/physiological in 49, access/system-related in 5, patient preference/adherence-related in 4, and undocumented in 58, indicating documentation/reassessment uncertainty rather than proven therapeutic inertia. Intermediate dosing should not be equated automatically with successful optimization. A clinically useful audit framework should therefore distinguish target dose, current dose history, clinically limited sub-target therapy, active titration, access barriers, patient preference/adherence, and unresolved sub-target therapy, while embedding timely laboratory review and named multidisciplinary responsibility into routine care.

## Figures and Tables

**Table 1:** Cohort characteristics according to active sacubitril/valsartan prescription.

Characteristic	Overall (*N* = 296)	ARNI Active (*n* = 239)	ARNI Not Active (*n* = 57)	*p* Value
Age, years	60.6 ± 12.5	61.0 ± 12.7	59.0 ± 11.5	0.241
Body mass index, kg/m^2^	28.8 ± 4.9	28.6 ± 5.0	29.9 ± 4.6	0.067
LVEF, %	31.2 ± 4.2	31.3 ± 4.2	30.7 ± 4.0	0.378
Systolic blood pressure, mmHg	109.8 ± 12.2	113.0 ± 10.5	96.4 ± 9.1	<0.001
eGFR, mL/min/1.73 m^2^	58.0 ± 18.7	61.9 ± 17.4	41.8 ± 14.9	<0.001
Serum potassium, mmol/L	4.67 ± 0.44	4.60 ± 0.39	4.99 ± 0.49	<0.001
Outpatient visits in previous year	4.8 ± 2.2	4.9 ± 2.2	4.4 ± 2.1	0.120
Male sex	237 (80.1%)	188 (78.7%)	49 (86.0%)	0.269
Saudi nationality	264 (89.2%)	214 (89.5%)	50 (87.7%)	0.641
Newly diagnosed HFrEF	98 (33.1%)	82 (34.3%)	16 (28.1%)	0.435
Ischemic etiology	195 (65.9%)	165 (69.0%)	30 (52.6%)	0.029
Diabetes mellitus	190 (64.2%)	152 (63.6%)	38 (66.7%)	0.759
Hypertension	219 (74.0%)	174 (72.8%)	45 (78.9%)	0.403
Coronary artery disease	219 (74.0%)	180 (75.3%)	39 (68.4%)	0.314
Chronic kidney disease	76 (25.7%)	43 (18.0%)	33 (57.9%)	<0.001
Atrial fibrillation	46 (15.5%)	39 (16.3%)	7 (12.3%)	0.545
Beta-blocker use	280 (94.6%)	226 (94.6%)	54 (94.7%)	1.000
MRA use	192 (64.9%)	163 (68.2%)	29 (50.9%)	0.020
SGLT2-inhibitor use	145 (49.0%)	128 (53.6%)	17 (29.8%)	0.002
Potassium ≥ 5.5 mmol/L	22 (7.4%)	10 (4.2%)	12 (21.1%)	<0.001
≥4 outpatient visits/year	209 (70.6%)	173 (72.4%)	36 (63.2%)	0.196

**Table 2:** Active sacubitril/valsartan status and prescribing delay according to index-year cohort.

Index-Year Cohort	Patients in Index-Year Cohort	ARNI Active	Prevalence	Days from Predefined ARNI Consideration Date to Prescription, Median [IQR]
2020	33	20	60.6%	45 [24–72]
2021	39	29	74.4%	46 [22–62]
2022	48	37	77.1%	33 [19–62]
2023	54	45	83.3%	27 [14–51]
2024	59	52	88.1%	24 [11–33]
2025	63	56	88.9%	14 [8–28]

Note: Time-to-prescription estimates include treated patients only and describe a documented prescribing-process interval. Index-year cohorts are mutually exclusive and do not represent repeated annual cross-sectional prevalence estimates.

**Table 3:** Sacubitril/valsartan initiation pathways.

Initiation Characteristic	Finding	Interpretation for Prescribing Practice
Newly diagnosed HFrEF among all patients	98 (33.1%)	Active prescription in 82/98 (83.7%)
Established HFrEF among all patients	198 (66.9%)	Active prescription in 157/198 (79.3%)
Switch from ACE inhibitor/ARB among treated	155 (64.9%)	Prior tolerance and 36-h washout required when switching from ACE inhibitor
ARNI initiation without documented prior ACE inhibitor/ARB exposure among treated	84 (35.1%)	No prior ACE inhibitor/ARB documented in the available EHR; the reason for this pathway was not adjudicated
Inpatient or immediate post-discharge initiation	75 (31.4%)	Initiation during a monitored transition of care
Outpatient initiation	164 (68.6%)	Monitoring depended on ambulatory workflow
Cardiology-led initiation	184 (77.0%)	Cardiologist initiated or explicitly directed treatment
Residence outside immediate catchment	88 (36.8%)	Potential travel and follow-up burden
Time from predefined ARNI consideration date to prescription	26 days [IQR 12–46]	Declined across calendar periods

**Table 4:** Selected physiological caution features and clinical context among patients without active sacubitril/valsartan (*n* = 57).

Clinical Context	*n* (%)
SBP < 100 mmHg (caution feature)	32 (56.1%)
eGFR < 30 mL/min/1.73 m^2^ (caution feature)	16 (28.1%)
Potassium ≥ 5.5 mmol/L (safety concern)	12 (21.1%)
At least one selected physiological caution feature	41 (71.9%)
Two or more selected physiological caution features	18 (31.6%)
No selected physiological caution feature	16 (28.1%)
Active ACE inhibitor or ARB	28 (49.1%)
ACE inhibitor/ARB among non-users without selected caution features	14/16 (87.5%)
Prior intolerance documented	8 (14.0%)
Patient declined or was reluctant	5 (8.8%)
Pending reassessment documented	9 (15.8%)

Note: Categories are not mutually exclusive. The three physiological variables are selected caution features, not absolute contraindications or a complete definition of treatment eligibility. ACE inhibitor/ARB use is treatment context rather than proof of ARNI suitability.

**Table 5:** Monitoring and care-process indicators in the dose-practice cohort (*n* = 221).

Care-Process Indicator	*n*/*N*	%	95% CI
Laboratory monitoring ordered within 14 days	180/221	81.4	75.8–86.0
Laboratory monitoring completed within 14 days	166/221	75.1	69.0–80.4
Follow-up encounter within 6 weeks	150/221	67.9	61.5–73.7
Pharmacist medication review within 90 days	139/221	62.9	56.4–69.0
Named titration plan/responsible clinician	145/221	65.6	59.1–71.6
At least one escalation attempt	139/221	62.9	56.4–69.0
Missed scheduled appointment during titration window	52/221	23.5	18.4–29.5
Temporary dose-strength supply problem	26/221	11.8	8.2–16.7
Telemedicine contact during titration window	34/221	15.4	11.2–20.7
Residence outside immediate catchment	81/221	36.7	30.6–43.2

Note: Percentages are based on the full dose-practice cohort (*n* = 221). Confidence intervals are 95% Wilson score intervals.

**Table 6:** Sacubitril/valsartan dose selection, escalation, and current sub-target therapy (*n* = 221).

Dose-Practice Outcome	*n*/*N*	%	95% CI
Starting dose			
24/26 mg twice daily	166/221	75.1	69.0–80.4
49/51 mg twice daily	55/221	24.9	19.6–31.0
Current dose			
24/26 mg twice daily	116/221	52.5	45.9–59.0
49/51 mg twice daily	68/221	30.8	25.1–37.1
97/103 mg twice daily	37/221	16.7	12.4–22.2
Highest documented dose			
24/26 mg twice daily	91/221	41.2	34.9–47.8
49/51 mg twice daily	82/221	37.1	31.0–43.6
97/103 mg twice daily	48/221	21.7	16.8–27.6
Dose escalation			
Any escalation attempt	139/221	62.9	56.4–69.0
Escalation within 90 days	134/221	60.6	54.1–66.8
Dose history among patients currently receiving 24/26 mg twice daily (*n* = 116)			
Never documented above 24/26 mg twice daily	91/116	78.4	70.1–85.0
Previously reached a higher dose and subsequently returned to 24/26 mg twice daily	25/116	21.6	15.0–29.9
Documented reason for current lowest-dose therapy (*n* = 116)			
Clinical/physiological limitation	49/116	42.2	33.6–51.3
Health-system/access limitation	5/116	4.3	1.9–9.7
Patient preference/adherence-related reason	4/116	3.4	1.3–8.5
No documented reason for non-escalation/reassessment	58/116	50.0	41.0–59.0

Note: Percentages for starting dose, current dose, highest documented dose, and escalation outcomes use the full dose-practice cohort (*n* = 221). Dose-history and reason-for-current-lowest-dose percentages use the current lowest-dose subgroup (*n* = 116). Confidence intervals are 95% Wilson score intervals. Dose-history categories and reason categories represent different dimensions and should not be summed together.

**Table 7:** Qualitative participant profile (*n* = 15).

Professional Group	*n*	Experience Range, Years	Principal Contribution to ARNI Pathway
Cardiologists	6	3–22	Clinical suitability, initiation, complex titration
Internists	3	5–16	Comorbidity management, referral, inpatient continuation
Clinical pharmacists	3	4–13	Reconciliation, washout, counseling, laboratory review
Cardiology/HF nurses	3	6–18	Education, appointment coordination, interim symptom contact

**Table 8:** Qualitative themes and explanatory contribution.

Theme	Illustrative CFIR Domains	Quantitative Phenomenon Explained	Interpretive Contribution
1. Normalization as foundational therapy	Evidence strength; relative priority; access	Higher active use and shorter prescribing delay in later index-year cohorts	Residual gaps were not primarily caused by disbelief in ARNI efficacy.
2. Dynamic clinical suitability judgment	Patient needs; adaptability; clinician capability	Selected physiological caution features common among non-users	Physiological suitability depended on symptoms, trajectory, and monitoring capacity.
3. Monitoring as hidden prescribing work	Work infrastructure; implementation process	Incomplete laboratory completion and follow-up	Ordering, reviewing, communicating, and acting were separate steps.
4. Multiple meanings of current low-dose therapy	Reflecting and evaluating; documentation	Among 116 lowest-dose patients: 49 clinical/physiological reasons, 5 access/system reasons, 4 patient preference/adherence reasons, and 58 no documented reason	Clinical limitation should be separated from prior de-escalation, access barriers, patient preference/adherence, and unresolved current sub-target therapy.
5. Multidisciplinary ownership and access	Teaming; engaging; available resources	Incomplete follow-up, missed visits, and diffuse responsibility within the pathway	Named responsibility and interim contact converted intention into action.

**Table 9:** Joint display of quantitative findings, qualitative explanations, and meta-inferences.

Quantitative Finding	Qualitative Explanation	Relationship	Meta-Inference
Later index-year cohorts: active therapy 60.6% to 88.9%; shorter prescribing delay	Clinicians described growing familiarity and normalization of ARNI use	Convergence	Calendar-period differences are consistent with pathway maturation, not repeated annual prevalence.
71.9% of non-users had ≥1 selected physiological caution feature	Clinicians used symptoms, trends, diuretic burden, and monitoring capacity in addition to single measurements	Expansion	Physiological measurements identify safety considerations but do not define eligibility by themselves.
75.1% completed laboratory reassessment; 67.9% had follow-up within six weeks	Monitoring was described as a multistep chain from ordering to review, communication, and action	Convergence	Post-initiation care can be lost at several sequential steps.
16.7% were currently at target dose; 21.7% had reached target at any point	Participants distinguished active titration, true clinical limitation, prior de-escalation, and unresolved current sub-target therapy	Expansion	Intermediate or low dosing should not automatically be classified as optimized.
Among 116 patients currently on the lowest dose, 91 had never been documented above it and 25 had previously reached a higher dose; reasons were 49 clinical/physiological, 5 access/system, 4 patient preference/adherence, and 58 undocumented	Clinicians distinguished physiological intolerance, prior escalation followed by de-escalation, access barriers, patient preferences, and absent reassessment plans	Convergence	Dose history and reason for current sub-target therapy should both be documented for meaningful audit.
23.5% missed an appointment and 36.7% lived outside the immediate catchment	Geographic and organizational friction accumulated through testing, travel, and communication	Complementarity	Access matters through the workflow rather than as a single isolated patient characteristic.

## Data Availability

The data supporting the findings of this study are available from the corresponding author upon reasonable request. Due to the qualitative nature of part of the study and the need to protect participant confidentiality, interview transcripts and other potentially identifiable data are not publicly available.

## Supplementary Materials

The following supporting information can be downloaded at: https://www.mdpi.com/article/10.3390/jcm15208001/s1, Supplementary File S1, completed Consolidated Criteria for Reporting Qualitative Research (COREQ) 32-item checklist; Supplementary File S2, semi-structured clinician interview guide; and Supplementary File S3, coding tree.

## Abbreviations

The following abbreviations are used in this manuscript:

ACE inhibitor	Angiotensin-converting enzyme inhibitor
ARB	Angiotensin receptor blocker
ARNI	Angiotensin receptor–neprilysin inhibitor
CFIR	Consolidated Framework for Implementation Research
COREQ	Consolidated Criteria for Reporting Qualitative Research
eGFR	Estimated glomerular filtration rate
EHR	Electronic health record
HF	Heart failure
HFrEF	Heart failure with reduced ejection fraction
IQR	Interquartile range
LVEF	Left-ventricular ejection fraction
MRA	Mineralocorticoid receptor antagonist
SBP	Systolic blood pressure
SGLT2	Sodium–glucose cotransporter-2
