# Supplementary material for: Real-World Sacubitril/Valsartan Adoption and Post-Initiation Dose Reassessment and Titration in Heart Failure with Reduced Ejection Fraction: An Explanatory Sequential Mixed-Methods Study from Northern Saudi Arabia
Source: J Clin Med. 2026 Oct 15;15(20):8001. doi: 10.3390/jcm15208001 (PMC13705730; doi:10.3390/jcm15208001)
Supplement: Supplementary file 1 [file jcm-15-08001-s001.zip › Supplementary File S1_COREQ Checklist.pdf]

Supplementary File S1. COREQ 32-Item Checklist

Real-World Sacubitril/Valsartan Adoption and Post-Initiation Dose Reassessment and Titration in Heart Failure with Reduced Ejection Fraction: An Explanatory Sequential Mixed-Methods Study from Northern Saudi Arabia

Domain	No.	COREQ item	Reported information	Manuscript location
Domain 1: Research team and reflexivity	1	Interviewer/facilitator	All interviews were conducted by Reham Olayan Alonayzan (R.O.A.), the first author.	Section 2.4.2, p. 6
	2	Credentials	R.O.A. was an MSc student in Clinical Pharmacy and researcher.	Section 2.4.2, p. 6
	3	Occupation	MSc student in Clinical Pharmacy and researcher at the time of data collection.	Section 2.4.2, p. 6
	4	Gender	Female.	Section 2.4.2, p. 6
	5	Experience and training	Before data collection, R.O.A. reviewed the interview guide and interviewing procedures with senior investigators and pilot-tested the guide; no additional formal qualitative-interviewing credential is claimed.	Section 2.4.2, p. 6
	6	Relationship established	No research relationship was established before recruitment; R.O.A. had no supervisory, line-management, or performance-appraisal relationship with participants. Incidental professional familiarity within the hospital was possible.	Section 2.4.2, p. 6
	7	Participant knowledge of interviewer	Participants were informed of the study purpose, the interviewer's clinical-pharmacy background, and that she was acting in a research rather than evaluative role.	Section 2.4.2, p. 6
	8	Interviewer characteristics/reflexivity	Potential assumptions arising from familiarity with HFrEF pharmacotherapy were acknowledged and addressed through reflexive memoing and multidisciplinary discussion.	Section 2.4.2 and 2.6, pp. 6–7
Domain 2: Study design	9	Methodological orientation and theory	Explanatory sequential mixed-methods design under a pragmatic paradigm; qualitative analysis used the Framework Method with CFIR-informed deductive sensitizing concepts and inductive coding.	Sections 2.1 and 2.5.2, pp. 3 and 7
	10	Sampling	Purposive sampling sought variation in profession, seniority, and inpatient versus ambulatory responsibilities among clinicians involved in HFrEF medication decisions.	Section 2.4.1, p. 6
	11	Method of approach	Potential participants were approached directly by the qualitative researcher.	Section 2.4.1, p. 6
	12	Sample size	Fifteen clinicians participated: six cardiologists, three internists, three clinical pharmacists, and three cardiology/HF nurses.	Sections 3.1 and 3.8, pp. 8 and 14
	13	Non-participation	Eighteen clinicians were approached; 15 participated and 3 did not. Reasons for non-participation were not recorded.	Sections 2.4.1 and 3.1, pp. 6 and 8
	14	Setting of data collection	Face-to-face interviews were conducted in a private hospital room at Al-Qurayyat General Hospital.	Section 2.4.3, p. 6
	15	Presence of non-participants	No non-participant was present during interviews.	Section 2.4.3, p. 6
	16	Description of sample	Professional group, experience, and principal contribution to the ARNI pathway are reported.	Section 3.8 and Table 7, p. 14
	17	Interview guide	A semi-structured guide informed by preliminary quantitative patterns, study objectives, and CFIR domains was pilot-tested. The full guide is supplied as Supplementary File S2.	Section 2.4.3, p. 6; Supplementary File S2
	18	Repeat interviews	No repeat interviews were conducted.	Section 2.4.3, p. 6
	19	Audio/visual recording	All interviews were audio-recorded after written informed consent.	Section 2.4.3, p. 6
	20	Field notes	Contextual and reflexive notes were recorded during and immediately after interviews to support interpretation and subsequent probing.	Sections 2.4.2–2.4.3, p. 6
	21	Duration	Interviews lasted approximately 28–46 minutes.	Section 2.4.3, p. 6
	22	Data saturation / analytic sufficiency	Recruitment continued until analytic sufficiency, defined as the point at which additional interviews no longer added materially new explanations of the principal quantitative findings.	Section 2.4.1, p. 6
	23	Transcripts returned	Full transcripts were not routinely returned to participants; participants could clarify or expand statements during the interview.	Section 2.4.3, p. 6
Domain 3: Analysis and findings	24	Number of data coders	Two researchers independently coded three information-rich transcripts; R.O.A. coded the remaining transcripts with regular review by the second qualitative analyst and senior study team.	Section 2.5.2, p. 7
	25	Description of coding tree	A hierarchical coding framework linking themes, subthemes, and key NVivo codes is provided.	Section 2.5.2, p. 7; Supplementary File S3
	26	Derivation of themes	Themes were developed iteratively using a hybrid deductive–inductive process, examining pattern, context, convergence, contradiction, and explanatory value.	Section 2.5.2, p. 7

	27	Software	NVivo (Lumivero, Denver, CO, USA) was used for transcript management, coding, retrieval, memoing, and framework matrices.	Section 2.5.2, p. 7
	28	Participant checking	Formal member checking of the final thematic structure was not undertaken. Participants could clarify and expand statements during interviews.	Sections 2.4.3 and 2.6, pp. 6–7
	29	Quotations presented	Illustrative quotations are presented with participant identifiers and professional roles.	Section 3.9, pp. 15–16
	30	Data and findings consistent	Qualitative findings are linked to quantitative findings through narrative integration, Table 9 joint display, and Figure 4.	Section 3.10, pp. 17–18
	31	Clarity of major themes	Five major themes are clearly described and summarized in Table 8.	Section 3.9 and Table 8, pp. 15–16
	32	Clarity of minor themes/diverse cases	Subthemes and negative/deviant cases are described, including clinically vulnerable patients successfully titrated and apparently stable patients with persistent lowest-dose therapy without clear reassessment.	Sections 2.5.2 and 3.9, pp. 7 and 15–16
