# Supplementary material for: Real-World Sacubitril/Valsartan Adoption and Post-Initiation Dose Reassessment and Titration in Heart Failure with Reduced Ejection Fraction: An Explanatory Sequential Mixed-Methods Study from Northern Saudi Arabia
Source: J Clin Med. 2026 Oct 15;15(20):8001. doi: 10.3390/jcm15208001 (PMC13705730; doi:10.3390/jcm15208001)
Supplement: Supplementary file 1 [file jcm-15-08001-s001.zip › Supplementary_File_S2_Interview Guide.pdf]

Supplementary File S2. Semi-Structured Clinician Interview Guide

Real-World Sacubitril/Valsartan Adoption and Post-Initiation Dose Reassessment and Titration in Heart Failure with Reduced Ejection Fraction: An Explanatory Sequential Mixed-Methods Study from Northern Saudi Arabia

Purpose and format

The guide was used to explore clinician perspectives on sacubitril/valsartan adoption, clinical suitability, initiation and switching, safety monitoring, dose reassessment and titration, patient and access barriers, documentation, and multidisciplinary responsibility in routine HFrEF care. It was pilot-tested before formal data collection for clarity, sequencing, and relevance. Questions were used flexibly, with probes adapted to each participant's professional role and experience.

Opening script

Thank you for taking part. We are interested in how sacubitril/valsartan is used in routine care for patients with HFrEF at this hospital, including how clinicians decide to start it, monitor it, reassess the dose, and manage practical barriers. There are no right or wrong answers. We are interested in your experience and views. With your permission, the interview will be audio-recorded and your responses will be reported anonymously.

Participant context prompts

Professional role; years of clinical experience; principal inpatient/outpatient responsibilities; involvement in HFrEF medication decisions; involvement in prescribing, medication reconciliation, laboratory monitoring, patient counseling, follow-up, or dose titration.

1. Perception of evidence and role of ARNI in HFrEF care

How do you view the role of sacubitril/valsartan in the routine management of patients with HFrEF in your service?

Suggested probes: How has your practice changed over the last few years?; How do guidelines influence whether you consider ARNI?; Are there situations in which you still prefer an ACE inhibitor or ARB?.

2. Clinical suitability and decision to initiate

When you consider starting sacubitril/valsartan, what patient factors most influence your decision?

Suggested probes: Blood pressure and symptoms; Kidney function and recent changes in creatinine/eGFR; Potassium; Congestion and diuretic burden; Frailty, dizziness, falls, or recent clinical instability; Ability to return for laboratory monitoring and follow-up.

3. Switching from ACE inhibitor/ARB and starting-dose selection

How do you manage switching a patient from an ACE inhibitor or ARB to sacubitril/valsartan and selecting the initial dose?

Suggested probes: ACE-inhibitor 36-hour washout; Previous ACE inhibitor/ARB dose and tolerance; Low blood pressure or kidney impairment; Differences between inpatient and outpatient initiation; Medication reconciliation.

4. Early laboratory monitoring and safety review

What usually happens after sacubitril/valsartan is started or the dose is increased?

Suggested probes: Who orders renal-function and potassium tests?; How soon are tests expected?; Who reviews the results?; How are abnormal results communicated?; What happens if the patient does not complete the test?.

5. Dose reassessment and titration

How are decisions made about increasing, maintaining, reducing, or temporarily stopping the dose?

Suggested probes: Usual timing of reassessment; What prevents escalation to 49/51 or 97/103 mg twice daily?; How do you distinguish a clinically limited dose from a dose that has simply not been revisited?; What happens after a previous higher dose is reduced?.

6. Responsibility and continuity of care

Who is responsible for ensuring that the dose is revisited after initiation?

Suggested probes: Is responsibility explicit or shared?; What happens when the plan only says 'follow in clinic'?; How do cardiologists, internists, pharmacists, and nurses communicate?; Who can act on laboratory results between scheduled visits?.

7. Appointments, geographic access, and missed follow-up

How do appointment availability, travel, or missed visits affect ARNI monitoring and dose reassessment?

Suggested probes: Patients living outside the immediate catchment; Availability of laboratory testing near home; Delays between laboratory testing and clinic review; Use of telephone or telemedicine contact; What happens after a missed appointment?.

8. Medication availability and pharmacy processes

Does medication or dose-strength availability affect initiation or titration in practice?

Suggested probes: Temporary shortages; Substitution between strengths; Communication between pharmacy and prescribers; Whether supply disruptions lead to prolonged lower dosing.

9. Patient understanding, preferences, and adherence

What patient-related factors make ARNI initiation or dose escalation easier or more difficult?

Suggested probes: Understanding of twice-daily dosing; Concerns about adverse effects; Reluctance to change from ACE inhibitor/ARB; Adherence and overall medication burden; Patient preference to remain on a lower dose.

10. Documentation of reasons for sub-target therapy

How well does the clinical record capture why a patient remains below the target dose?

Suggested probes: Clinical/physiological limitation; Access or health-system limitation; Patient preference/adherence; No documented reason; Whether the next reassessment date and responsible clinician are recorded.

11. Multidisciplinary roles

What roles do pharmacists and nurses currently play, or could they play, in improving ARNI follow-up and titration?

Suggested probes: Medication reconciliation and ACE-inhibitor washout verification; Laboratory review; Patient counseling; Appointment reminders; Interim follow-up; Escalation prompts or protocol-supported titration.

12. Improvement priorities

If you could change the local pathway to improve ARNI prescribing and dose reassessment, what would you change first?

Suggested probes: Standardized initiation order sets; Automatic laboratory reminders; Registry or list of patients below target dose; Named titration ownership; Rapid-access appointments; Pharmacist- or nurse-supported interim contacts; Any additional issue not yet discussed.

Closing question

Is there anything else about sacubitril/valsartan prescribing, monitoring, dose reassessment, or the organization of heart-failure care that you think is important and that we have not discussed?
