# Supplementary material for: Real-World Sacubitril/Valsartan Adoption and Post-Initiation Dose Reassessment and Titration in Heart Failure with Reduced Ejection Fraction: An Explanatory Sequential Mixed-Methods Study from Northern Saudi Arabia
Source: J Clin Med. 2026 Oct 15;15(20):8001. doi: 10.3390/jcm15208001 (PMC13705730; doi:10.3390/jcm15208001)
Supplement: Supplementary file 1 [file jcm-15-08001-s001.zip › Supplementary_File_S3_Coding Tree.pdf]

Supplementary File S3. Qualitative Coding Tree and NVivo Node Structure

Real-World Sacubitril/Valsartan Adoption and Post-Initiation Dose Reassessment and Titration in Heart Failure with Reduced Ejection Fraction: An Explanatory Sequential Mixed-Methods Study from Northern Saudi Arabia

Analytic approach. The coding structure was developed iteratively using the Framework Method. CFIR domains were used as sensitizing concepts, while inductive coding captured clinically and organizationally specific issues emerging from the interviews. The hierarchy below summarizes the final thematic structure used to organize NVivo coding and framework matrices.

Root category	Major theme	Subtheme	Representative NVivo codes/nodes	Illustrative CFIR domains
Root: ARNI implementation pathway	Theme 1. Sacubitril/valsartan normalized as foundational therapy	1.1 Guideline endorsement and evidence strength	Guideline awareness; perceived evidence strength; relative priority of ARNI	Intervention/evidence; individuals
		1.2 Formulary access and growing familiarity	Formulary availability; availability of dose strengths; clinician familiarity; repeated exposure	Inner setting; available resources
		1.3 Shift from selective to routine consideration	Earlier conservative/selective use; normalization; proactive consideration; routine switching	Implementation process; relative priority
Root: ARNI implementation pathway	Theme 2. Clinical suitability as dynamic safety judgment	2.1 Blood pressure and symptomatic tolerance	Low SBP; dizziness; orthostasis; falls; symptom burden	Individuals; patient needs
		2.2 Renal function and trajectory	eGFR; creatinine trend; chronic kidney disease; recent acute kidney injury	Individuals; adaptability
		2.3 Potassium and biochemical safety	Hyperkalemia; repeat potassium; biochemical monitoring	Individuals; patient needs
		2.4 Overall vulnerability and clinical context	Frailty; congestion; diuretic burden; recent instability; multimorbidity	Individuals; complexity
		2.5 Monitoring capacity as part of suitability	Ability to return; access to laboratory testing; confidence in follow-up; ability to review results	Outer setting; work infrastructure
Root: ARNI implementation pathway	Theme 3. Monitoring as the hidden work of prescribing	3.1 Ordering and completing laboratory tests	Laboratory orders; test completion; delays; missed testing	Implementation process; work infrastructure
		3.2 Reviewing results and acting	Result review; interpretation; authorization of next dose; escalation/reduction decision	Implementation process; responsibility
		3.3 Communicating with the patient	Phone contact; counseling; communicating abnormal results; instructions after testing	Engaging; implementation process
		3.4 Follow-up continuity	Early follow-up; appointment availability; missed visits; delayed clinic review	Outer setting; implementation process
		3.5 Named responsibility versus diffuse ownership	Named titration plan; unclear ownership; 'follow in clinic'; handoff gaps	Teaming; implementation process
Root: ARNI implementation pathway	Theme 4. Current low-dose therapy had multiple meanings	4.1 Clinical/physiological limitation	Symptomatic hypotension; renal dysfunction; hyperkalemia; frailty/orthostatic symptoms	Individuals; patient needs
		4.2 Previous titration followed by de-escalation	Previously reached higher dose; dose reduction; restart at lower dose; recurrent intolerance	Adaptability; implementation process
		4.3 Health-system/access limitation	Appointment delays; travel burden; laboratory access; service capacity	Outer setting; inner setting
		4.4 Patient preference/adherence-related limitation	Reluctance; adherence; twice-daily dosing; medication burden; preference for lower dose	Individuals; patient needs
		4.5 Unresolved low-dose persistence	No reassessment; no documented reason; absent review date; passive persistence	Reflecting/evaluating; documentation
Root: ARNI implementation pathway	Theme 5. Multidisciplinary ownership and access determined whether intention became action	5.1 Physician/cardiologist decision authority	Initiation decision; final titration decision; complex clinical review	Teaming; individuals
		5.2 Clinical pharmacist continuity functions	Medication reconciliation; ACE-inhibitor washout; laboratory review; counseling; escalation prompts	Teaming; engaging
		5.3 Nursing coordination	Patient education; appointment coordination; interim symptom contact; reminders	Teaming; engaging

		5.4 Geographic and travel barriers	Outside catchment; rural travel; delayed testing; transport burden	Outer setting
		5.5 Medication supply and dose-strength availability	Temporary shortages; strength substitution; pharmacy-prescriber communication	Available resources
		5.6 Enabling service solutions	Standardized order set; automatic laboratory reminders; below-target registry; rapid-access clinic; telemedicine	Implementation process; work infrastructure
